# Lifespan analysis of brain development, gene expression and behavioral phenotypes in the Ts1Cje, Ts65Dn and Dp(16)1/Yey mouse models of Down syndrome

**DOI:** 10.1242/dmm.031013

**Published:** 2018-06-12

**Authors:** Nadine M. Aziz, Faycal Guedj, Jeroen L. A. Pennings, Jose Luis Olmos-Serrano, Ashley Siegel, Tarik F. Haydar, Diana W. Bianchi

**Affiliations:** 1 Department of Anatomy and Neurobiology, Boston University School of Medicine, Boston, MA 02118, USA; 2 National Human Genome Research Institute, National Institutes of Health, Bethesda, MD 20892, USA; 3 Center for Health Protection, National Institute for Public Health and the Environment, 3720 BA Bilthoven, The Netherlands

**Keywords:** Down syndrome, Ts65Dn, Ts1Cje, Dp(16)1/Yey, Developmental disorders, Lifespan analysis, Brain development

## Abstract

Down syndrome (DS) results from triplication of human chromosome 21. Neuropathological hallmarks of DS include atypical central nervous system development that manifests prenatally and extends throughout life. As a result, individuals with DS exhibit cognitive and motor deficits, and have delays in achieving developmental milestones. To determine whether different mouse models of DS recapitulate the human prenatal and postnatal phenotypes, here, we directly compared brain histogenesis, gene expression and behavior over the lifespan of three cytogenetically distinct mouse models of DS: Ts1Cje, Ts65Dn and Dp(16)1/Yey. Histological data indicated that Ts65Dn mice were the most consistently affected with respect to somatic growth, neurogenesis and brain morphogenesis. Embryonic and adult gene expression results showed that Ts1Cje and Ts65Dn brains had considerably more differentially expressed (DEX) genes compared with Dp(16)1/Yey mice, despite the larger number of triplicated genes in the latter model. In addition, DEX genes showed little overlap in identity and chromosomal distribution in the three models, leading to dissimilarities in affected functional pathways. Perinatal and adult behavioral testing also highlighted differences among the models in their abilities to achieve various developmental milestones and perform hippocampal- and motor-based tasks. Interestingly, Dp(16)1/Yey mice showed no abnormalities in prenatal brain phenotypes, yet they manifested behavioral deficits starting at postnatal day 15 that continued through adulthood. In contrast, Ts1Cje mice showed mildly abnormal embryonic brain phenotypes, but only select behavioral deficits as neonates and adults. Altogether, our data showed widespread and unexpected fundamental differences in behavioral, gene expression and brain development phenotypes between these three mouse models. Our findings illustrate unique limitations of each model when studying aspects of brain development and function in DS. This work helps to inform model selection in future studies investigating how observed neurodevelopmental abnormalities arise, how they contribute to cognitive impairment, and when testing therapeutic molecules to ameliorate the intellectual disability associated with DS.

This article has an associated First Person interview with the first author of the paper.

## Abstract (precis)

**Editor's choice:** Comparison of corticogenesis, gene expression and behavior in three mouse models of Down syndrome revealed major differences between the models as well as significant limitations in each strain for understanding neurobiological changes in the human phenotype.

## INTRODUCTION

Down syndrome (DS) is a developmental disorder caused by triplication of human chromosome 21 (HSA21). Approximately 550 genes are located on HSA21, 222 of which encode proteins, while 325 encode microRNAs, long-noncoding RNAs and other regulatory elements (Gupta et al., 2016). DS is the most common live-born autosomal aneuploidy, with an incidence of 1 in 792 live births (de Graaf et al., 2015).

The neuropathological consequences of trisomy 21 begin \during fetal development with decreased cell division and increased apoptosis that together lead to hypoplasia of the hippocampus, neocortex and cerebellum (Bahado-Singh et al., 1992; Contestabile et al., 2007; Guidi et al., 2008; Guihard-Costa et al., 2006; Larsen et al., 2008; Petit et al., 1984; Rotmensch et al., 1997; Weitzdoerfer et al., 2001; Winter et al., 1998; Wisniewski et al., 1984). Brains from fetuses with DS also show a decrease in cellular migration and neurotransmitter levels (Whittle et al., 2007). After birth, brain morphology continues to diverge compared with typically developing individuals (Lott, 2012). In particular, cortical layer thickness, dendritic branching, synapse formation, brain size and overall brain weight are all reduced (Becker et al., 1991, 1986; Golden and Hyman, 1994; Pinter et al., 2001b; Ross et al., 1984; Schmidt-Sidor et al., 1990; Takashima et al., 1981; Wisniewski, 1990; Wisniewski et al., 1984). Delayed and altered myelination of white matter tracts has also been reported (Olmos-Serrano et al., 2016a; Wisniewski and Schmidt-Sidor, 1989). Presumably as a result of these neuroanatomical deficits, altered cognitive development is also observed in infants and children with DS. Intellectual and physical developmental delays eventually lead to a progressive decline in intellectual quotient, delayed language acquisition, and altered hippocampal-dependent explicit and spatial memory (Fidler and Nadel, 2007; Gibson et al., 1988; Nadel, 2003; Ohr and Fagen, 1994; Pennington et al., 2003; Vicari, 2004; Vicari et al., 2004, 2013; Wishart, 1993). By adulthood, brains of individuals with DS show a 24% reduction in size, with a decrease in volume of the cerebellum (−33%), hippocampus (−27%) and frontal cortex (−17%) (Coyle et al., 1986; Jernigan et al., 1993; Pinter et al., 2001a,b; Raz et al., 1995; Teipel et al., 2004, 2003; White et al., 2003; Wisniewski, 1990).

To date, treatment options for people with DS have been limited because human studies have been insufficient in generating longitudinal molecular, biochemical and functional data to elucidate specific, targetable mechanisms underlying DS phenotypes (de Wert et al., 2016). To address this, many genetically heterogeneous mouse models of DS have been generated to identify the mechanisms underlying the developmental changes in DS, and to provide a tractable approach for designing and testing potential therapeutic strategies. Although these studies on different DS models have significantly advanced our understanding, a lack of standardized testing paradigms has resulted in conflicting data and hindered direct comparisons of DS-related murine phenotypes (Das and Reeves, 2011; Gupta et al., 2016; Haydar and Reeves, 2012; Herault et al., 2017; Moore and Roper, 2007; Starbuck et al., 2014). Thus, there is a substantial unmet need for a detailed intraspecies comparative analysis that links the triplicated HSA21 orthologs with developmental sequelae in trisomy models exhibiting different DS phenotypes.

Here, we compare gene expression, brain histopathology and behavior in three cytogenetically distinct mouse models of DS: Ts1Cje, Ts65Dn and Dp(16)1/Yey mice. These three mouse models exploit the synteny that exists between HSA21 and the distal portion of mouse chromosome 16 (MMU16). Therefore, while all three models have large segments of triplicated HSA21-orthologous genes on MMU16, each was engineered using distinct methodology, resulting in different numbers of triplicated genes. The Ts1Cje mouse model was generated via a reciprocal translocation of the distal portion of MMU16 onto the telomeric region of MMU12 (Fig. S1) (Sago et al., 1998). This created an elongated MMU12 carrying an additional dose of 71 HSA21 orthologs, but also led to the monosomy of seven telomeric genes (Duchon et al., 2011). Despite the lack of nonorthologous triplicated genes in this model, a smaller triplication segment and loss of a functional copy of superoxide dismutase 1 has led to less frequent study of Ts1Cje mice. The Ts65Dn mouse was generated by cesium irradiation that induced a reciprocal translocation of the most distal portion of MMU16 onto a separate marker chromosome containing the centromeric portion of MMU17 (Davisson et al., 1990) (Fig. S1). Because this triplication is carried as an additional freely segregating chromosome, the Ts65Dn mouse uniquely models the aneuploidy observed in 95% of individuals with DS (Shin et al., 2010). The triplicated segment consists of ∼104 HSA21 orthologs as well as 60 centromeric MMU17 genes that are not triplicated in humans with DS (Duchon et al., 2011). Of these 60 unrelated genes, ∼35 code for proteins (Duchon et al., 2011). Despite this genetic dissimilarity to people with DS, Ts65Dn has historically been the most widely used trisomic mouse model. More recently, the Dp(16)1/Yey mouse model was generated using Cre-mediated recombination to duplicate the entire 23.3 Mb segment of HSA21 orthologs (∼119 genes), adding them onto the distal portion of one of the endogenous MMU16 chromosomes (Li et al., 2007) (Fig. S1). As this model contains the largest number of triplicated HSA21 orthologs and lacks any perturbation of unrelated genes compared with Ts1Cje and Ts65Dn mice, Dp(16)1/Yey mice should, in theory, have the most similar murine representation of the phenotypes seen in people with DS.

In this study, we used a standardized battery of tests to examine these three mouse strains side by side at three different life stages to eliminate confounding variables arising from experimental design or experimenter bias. We provide evidence of unexpectedly unique phenotypes in each model across the lifespan, and compare our findings with human studies. Furthermore, we provide an objective baseline format to aid in the evaluation of future mouse model(s) and to test the effects of potential therapeutic interventions.

## RESULTS

We compared gene expression, corticogenesis and behavioral aberrations in Ts1Cje, Ts65Dn and Dp(16)1/Yey mice at embryonic day (E) 15.5, postnatal days (P) 0-21, and in adulthood. The data show that Ts65Dn mice exhibited consistent histogenesis and behavioral deficits at every age. While Ts1Cje and Dp(16)1/Yey mice lacked the expected DS-related brain changes at E15.5, they manifested atypical cellular and behavioral phenotypes at different postnatal ages. Generally, gene expression changes were more pronounced in embryonic and adult Ts65Dn and Ts1Cje brains, and the lowest numbers of differentially expressed (DEX) genes were found in Dp(16)1/Yey brains at both embryonic and adult time points. Analyses of gene identity, function, regional expression and contribution to pathway perturbations demonstrated profound differences between the three models. A comprehensive summary of the results is presented in Table 1.

**Table 1.**
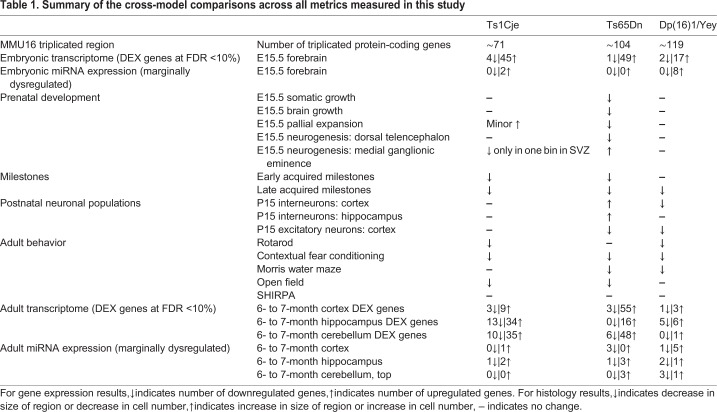
**Summary of the cross-model comparisons across all metrics measured in this study**

### Embryonic brain gene expression studies

Embryonic gene expression datasets were previously published but re-analyzed here using different false discovery rate (FDR) cut-offs and pathway analysis databases (Guedj et al., 2016).

#### DEX genes at various FDRs

We compared the number of DEX genes at three different stringencies (FDR<5%, <10% and <20%, Table S1). At all stringencies, Dp(16)1/Yey forebrains had the lowest number of DEX genes compared with Ts65Dn and Ts1Cje forebrains. The number of upregulated DEX genes was higher than the number of downregulated DEX genes in all three models, and most upregulated genes mapped to the triplicated region in each strain (Table S2A,B).

**Fig. 1.**
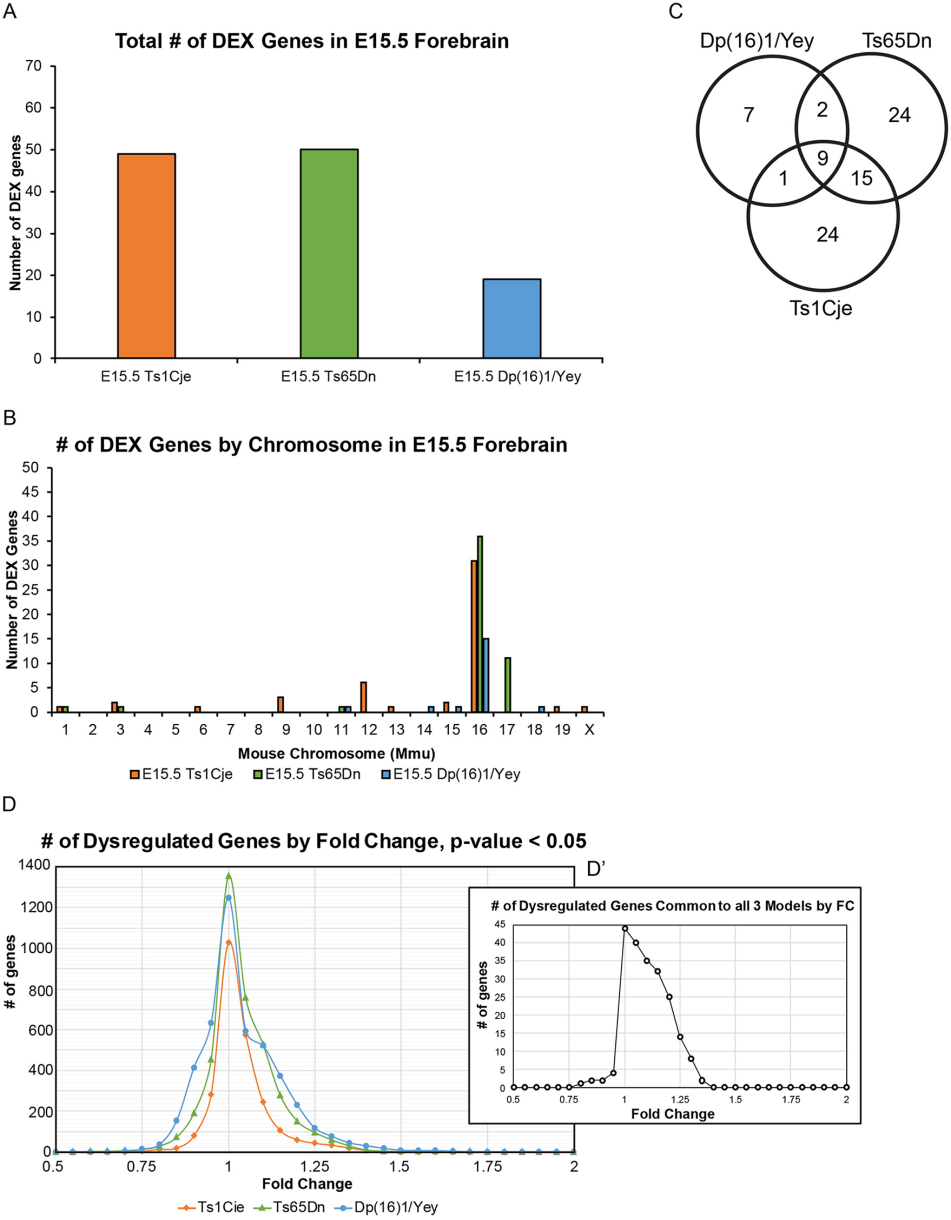
**Number of DEX genes in Ts1Cje, Ts65Dn and Dp(16)1/Yey embryonic forebrains.** Analysis of differentially expressed (DEX) genes in E15.5 forebrains of Ts1Cje mice (*n*=5 trisomic mice, *n*=5 euploid littermates); Ts65Dn mice (*n*=5 trisomic mice, *n*=6 euploid littermates); and Dp(16)1/Yey mice (*n*=6 trisomic mice, *n*=6 euploid littermates). A Benjamini-Hochberg FDR cut-off <10% was used to determine which genes are classified as DEX. (A) Overall number of DEX genes in each model. Ts1Cje and Ts65Dn mice display approximately double the number of DEX genes as Dp(16)1/Yey mice. (B) Number of DEX genes by chromosome in each model. (C) Venn diagram showing the number of common DEX genes among the models. (D) Distribution of dysregulated genes by fold change (FC), showing that the majority of dysregulated genes have small magnitude FCs that lie between 0.75 and 1.25. Relative gene expression in trisomic animals compared with their euploid littermates was deemed significant at *P*<0.05. (D′) Distribution of dysregulated genes that are common to all three models. The majority of dysregulated genes in common cluster between 0.75 and 1.25 FC.

At an FDR<10%, Ts1Cje and Ts65Dn forebrains had a comparable number of DEX genes (49 and 50, respectively) when compared with their euploid littermates (Fig. 1A; Table S2B). Almost all DEX genes were upregulated and over 50% of these DEX genes mapped to the MMU16 trisomic region (31 and 36 DEX genes for Ts1Cje and Ts65Dn mice, respectively) (Fig. 1C; Table S2B). Nine DEX genes in Ts65Dn forebrains mapped to the trisomic MMU17 centromeric region, and four DEX genes in Ts1Cje forebrains mapped to the monosomic region on MMU12 (Fig. 1B; Table S2B). The remaining 14 DEX genes in Ts1Cje forebrains, as well as five genes in Ts65Dn forebrains, mapped to other unaffected chromosomes without any specific clustering (Fig. 1B; Table S2B). Even though Dp(16)1/Yey mice contained the largest number of triplicated genes, only 19 DEX genes were found in Dp(16)1/Yey embryonic forebrains compared with their euploid littermates (Fig. 1A; Table S2A,B). Fifteen of these 19 genes mapped to the MMU16 trisomic region (Fig. 1B; Table S2B). In contrast to the other two mouse models, only four DEX genes mapped to unaffected chromosomes (Fig. 1B; Table S2B).

When the three mouse models were compared, Ts1Cje and Ts65Dn mice shared 24 DEX genes, all of which except *Rfx5* mapped to the MMU16 triplicated region (Fig. 1C; Table S2B). Dp(16)1/Yey embryonic brains shared 11 DEX genes with Ts65Dn and 10 DEX genes with Ts1Cje, all of which also mapped to the MMU16 triplicated region (Fig. 1C; Table S2B). Only nine DEX genes (*Urb1*, *Synj1*, *Son*, *Donson*, *Cryzl1*, *Ttc3*, *Dyrk1a*, *Psmg1* and *Brwd1*) were found to be common among all three models (Fig. 1C; Table S2B).

#### Analysis of non-MMU16 aneuploid genes in the Ts65Dn and Ts1Cje models

In addition to the MMU16 triplicated genes, Ts65Dn mice carry a triplication of ∼35 protein-coding MMU17 genes that are not orthologous to HSA21 genes (Fig. S1) (Duchon et al., 2011). Our microarray studies found that nine of these genes (*Scaf8*, *Tfb1m*, *Arid1b*, *Tmem242*, *Serac1*, *Gtf2h5*, *Tulp4*, *Rps6ka2* and *Fgfr1op*) were significantly upregulated in the Ts65Dn embryonic forebrain compared with euploid littermates (Table S3).

Ts1Cje mice also contain a monosomy of seven genes on MMU12 (Fig. S1) (Duchon et al., 2011). Three of these genes (*Itgb8*, *Sp4* and *Sp8*) were significantly downregulated, whereas *Dnah11* was significantly and markedly upregulated (Table S4).

#### Comparison of genome-wide effects in Ts1Cje, Ts65Dn and Dp(16)1/Yey embryonic forebrains

The differences between trisomic mice of each strain and their euploid littermates were subtle. Using a stringency of FDR<10% yielded a very low number of DEX genes that mostly clustered within the MMU16 trisomic region of each model. Therefore, in order to analyze the genome-wide expression effects in Ts1Cje, Ts65Dn and Dp(16)1/Yey embryonic forebrains, we set a raw *P*-value threshold of *P*<0.05 to determine which genes exhibited statistically significant alterations. We then determined the number of significantly altered genes at various fold change (FC) values from 0.5 to 2.0 (Fig. 1D). The highest number of altered genes, and the highest number of genes in common between the three models, were found within an FC range of 0.8 to 1.25 (Fig. 1D,D′). This indicated that the majority of dysregulated genes in trisomic forebrains from each of the models exhibited small FC values. As it is known that small FCs in gene expression are a hallmark of DS (Dauphinot et al., 2005; Laffaire et al., 2009; Lyle et al., 2004; Vilardell et al., 2011), we conducted a gene set enrichment analysis (GSEA) to include all misexpressed genes in an unbiased manner.

Consistent with our observation of the low number of overlapping DEX genes between the Ts1Cje, Ts65Dn and Dp(16)1/Yey models, only interferon signaling (upregulated) and amino acid transmembrane transporter activity (downregulated) were consistently altered in all three models at E15.5 (Table S5).

In Ts1Cje embryonic forebrains, gene sets associated with cell cycle regulation (metaphase), spindle pole and kinetochore assembly, DNA repair, JAK-STAT signaling, and Plk1 and Aurora B pathways were highly upregulated. In contrast, cell cycle genes were downregulated in both Ts65Dn and Dp(16)1/Yey embryonic forebrains, while spindle pole and kinetochore assembly, DNA repair and Plk1 pathways were downregulated only in Dp(16)1/Yey embryonic forebrains. Also, in both Ts1Cje and Ts65Dn embryos, amine-derived hormones and peptidyl tyrosine phosphorylation were upregulated, and the integrin 3 pathway was downregulated (Table S5).

In Ts65Dn embryonic forebrains, gene sets associated with synaptogenesis and NFAT signaling were significantly upregulated whereas ribonuclease activity and antigen binding were significantly downregulated. Ts65Dn and Dp(16)1/Yey embryonic forebrains shared more downregulated pathways, including cell cycle, helicase activity, glycosaminoglycan metabolism, major histocompatibility complex (MHC) class II antigen presentation, E2F protein pathway, SMAD2/3 pathway and DAG-IP3 signaling pathways. Cytokine binding, and GATA3 and NO2IL12 pathways were upregulated in both of these mouse models (Table S5).

In Dp(16)1/Yey embryonic forebrains, cytokine binding, G-protein signaling, extracellular matrix (NABA collagens), gap junction assembly and calcium channel activity were all upregulated. On the other hand, genes related to the ribosome, Golgi complex, endoplasmic reticulum and mitochondrial structures, ERK1/2 and WNT signaling pathways were downregulated (Table S5).

Notably, despite the low number of DEX genes, GSEA data indicated that Dp(16)1/Yey embryonic forebrains had the largest number of altered functional pathways compared with Ts1Cje and Ts65Dn mice (Table S5).

#### Dysregulated pathways and cellular processes

As mentioned above, using a stringency of FDR<10% yielded a very low number of DEX genes that could not be subjected to many of the common bioinformatics platforms. Relaxing statistical criteria to perform GSEA created a need for a complementary approach that incorporated some form of statistical interrogation. Therefore, we generated another gene category that we designated as ‘marginally dysregulated genes’ (MDGs) (as opposed to DEX) genes. In order to generate this list of MDGs, we gated the significantly altered genes (*P*<0.05) at FC<0.8 and FC>1.2 (at least ±20% change in expression level). These values were within the tails of the bell-shaped distribution of significantly dysregulated genes (Fig. 1D). The benefit of the MDG list was that it was unbiased and had a threshold based on statistical significance, but was less stringently gated than with an FDR<10%.

In contrast to DEX genes, Dp(16)1/Yey forebrain had the largest number of MDGs (229 upregulated and 36 downregulated) compared with Ts65Dn (150 upregulated and 28 downregulated) and Ts1Cje forebrains (59 upregulated and 12 downregulated) (Table S6). Importantly, when this MDG list was used for pathway analysis with the Database of Annotation, Visualization and Integrated Discovery (DAVID), similarities with GSEA in affected pathways were apparent. In particular, DAVID analysis also showed only a few commonly dysregulated pathways between models, including (1) upregulation of interferon signaling and immune response in all three mouse models; (2) upregulation of olfactory signaling in both Ts65Dn and Dp(16)1/Yey mice; (3) upregulation of JAK-STAT signaling in both Ts1Cje and Dp(16)1/Yey mice; and (4) upregulation in genes related to locomotor behavior in Ts1Cje and Ts65Dn mice (Tables S7 and S8). In addition to these common pathways, each mouse model exhibited unique pathway changes as follows: (1) Ts65Dn embryonic forebrains showed dysregulation of genes important for axonogenesis, cerebellar granule cell precursor proliferation and neural crest development; and (2) Dp(16)1/Yey showed upregulation of extracellular matrix genes and keratinocyte development, and downregulation of genes responsible for somatic stem cell maintenance, regulation of sequence-specific DNA binding, ribosomes and ribosomal structure, response to hypoxia and response to estradiol (Table S7).

#### Comparison of miRNA expression in Ts1Cje, Ts65Dn and Dp(16)1/Yey embryonic forebrain

MicroRNAs (miRNAs) are short noncoding RNAs that play a crucial role in gene expression through silencing and post-transcriptional regulation (Bartel, 2009; Bushati and Cohen, 2007). Several studies have analyzed the roles of specific HSA21-encoded miRNAs in different disease contexts, but global miRNA expression in fetal brains with DS has not yet been investigated (Elton et al., 2010; Gupta et al., 2016; Xu et al., 2013). Additionally, an miRNA expression study in adult Ts65Dn brains showed genome-wide miRNA dysregulation and left an open question about miRNA status in embryonic brains of these mice along with the Ts1Cje and Dp(16)1/Yey mice (He et al., 2013). We therefore assessed global miRNA representation within the MDG list in each model. The largest number of marginally dysregulated miRNAs (eight miRNAs) was found in the Dp(16)1/Yey embryonic forebrain (Table S9). In contrast, no miRNAs were dysregulated in the Ts65Dn embryonic forebrain and only two miRNAs were upregulated in the Ts1Cje embryonic forebrain (Table S9).

#### Quantitative RT-PCR validation of embryonic microarray findings

We validated four genes from the microarrays by TaqMan-based quantitative reverse-transcription polymerase chain reaction (qRT-PCR). These genes are as follows: (1) *Hspa13* (three copies in Dp(16)1/Yey only), (2) *App* (three copies in Ts65Dn and Dp(16)1/Yey), (3) *Ttc3* (three copies in all models) and (4) *Rfx5* (two copies in all models). In line with gene dosage, *Hspa13* was significantly overexpressed in Dp(16)1/Yey E15.5 forebrains in both microarray (FC=1.37, *P*=0.0016) and qRT-PCR (FC=1.44, *P*<0.05) experiments. *App* was unchanged in Ts1Cje brains, upregulated in Ts65Dn brains by both methods (microarrays: FC=1.25, *P*<0.01; qRT-PCR: FC=1.66, *P*<0.05), and upregulated only by qRT-PCR in Dp(16)1/Yey brains (microarrays: FC=1.23, n.s.; qRT-PCR: FC=1.54, *P*<0.05). *Ttc3* was significantly upregulated in all three models by both methods (Table S10). Lastly, *Rfx5* (present in two copies in all models) was consistently downregulated by microarrays and qRT-PCR in all three models (Table S10). These qRT-PCR results confirm the validity of the gene expression microarray findings (Table S10).

### Neuroanatomy and neurogenesis

#### Body and brain growth measurements in DS model embryos

**Fig. 2.**
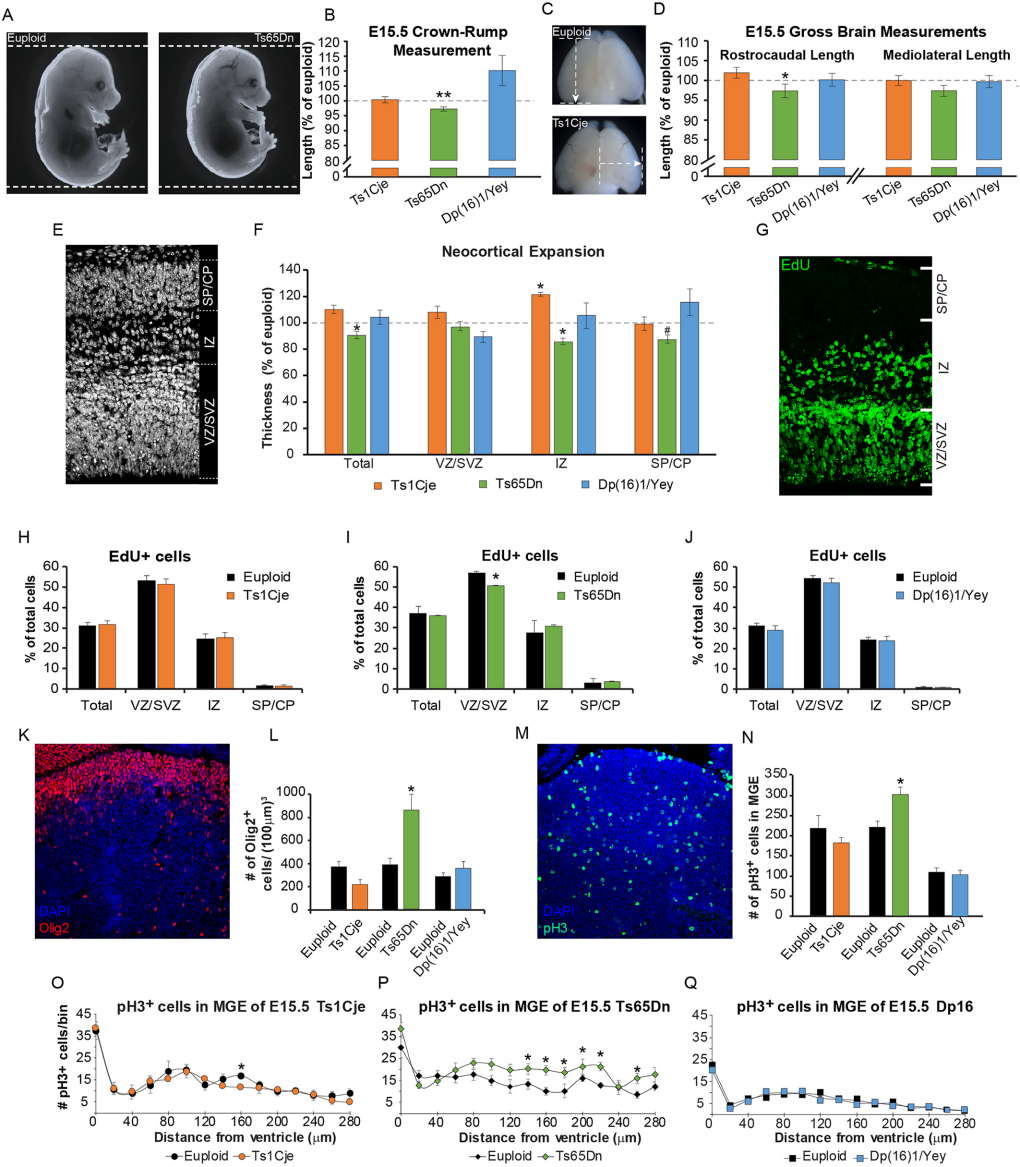
**Embryonic somatic growth, brain development, and neurogenesis in Ts1Cje, Ts65Dn and Dp(16)1/Yey mice.** All images and data are generated at the level of the future somatosensory cortex. Data are mean±s.e.m., *P<0.05, **P<0.01. (A) Representative images of euploid and Ts65Dn embryos at E15.5. (B) Quantification of body length in Ts1Cje, Ts65Dn and Dp(16)1/Yey embryos, showing only a decrease in Ts65Dn body length. Mice used: (1) Ts1Cje strain (*n*=13 trisomic mice, *n*=11 euploid littermates); (2) Ts65Dn strain (*n*=7 trisomic mice, *n*=20 euploid littermates); (3) Dp(16)1/Yey strain (*n*=26 trisomic mice, *n*=19 euploid littermates). (C) Representative images displaying the rostrocaudal (top) and mediolateral (bottom) measurements used to assess gross brain size at E15.5. (D) Gross brain measurements in Ts1Cje, Ts65Dn and Dp(16)1/Yey mice, showing that only Ts65Dn embryonic forebrains have a decreased rostrocaudal length. Mice used: (1) Ts1Cje strain (*n*=13 trisomic mice, *n*=11 euploid littermates); (2) Ts65Dn strain (*n*=7 trisomic mice, *n*=20 euploid littermates); (3) Dp(16)1/Yey strain (*n*=26 trisomic mice, *n*=19 euploid littermates). (E) Representative image showing the dorsal pallium in E15.5 brain. Dashed lines demarcate the different layers of the germinal zone: ventricular/subventricular zones (VZ/SVZ), intermediate zone (IZ) and subplate/cortical plate (SP/CP). (F) Measures of neocortical expansion in Ts1Cje, Ts65Dn and Dp(16)1/Yey forebrains as a percentage of those of their respective euploid littermates. Ts65Dn embryos show a decrease in overall pallial thickness, as well as thickness of the IZ and SP/CP (^#^*P*=0.10). Ts1Cje embryos show an increase in the size of the IZ that is not reflected in any other layer or in overall thickness. Dp(16)1/Yey embryos show no change. Mice used: (1) Ts1Cje strain (*n*=6 trisomic mice, *n*=6 euploid littermates); (2) Ts65Dn strain (*n*=9 trisomic mice, *n*=9 euploid littermates); (3) Dp(16)1/Yey strain (*n*=11 trisomic mice, *n*=10 euploid littermates). (G) Representative image showing EdU staining (green) in the dorsal pallium. Again, the layers of the dorsal germinal zone are demarcated. (H) Ts1Cje embryos show no change in the percentage of EdU^+^ cells by layer in the dorsal pallium compared with euploid littermates. (I) Ts65Dn embryos show a decrease in the percentage of EdU^+^ cells only in the VZ/SVZ of the dorsal pallium compared with euploid littermates. (J) Dp(16)1/Yey embryos show no change in the percentage of EdU^+^ cells by layer in the dorsal pallium compared with euploid littermates. (K) Representative image showing OLIG2 (red) staining in the medial ganglionic eminence (MGE) of the ventral germinal zone at E15.5. Cell nuclei are stained with DAPI (blue). Mice used in H-K: (1) Ts1Cje strain (*n*=6 trisomic mice, *n*=6 euploid littermates); (2) Ts65Dn strain (*n*=9 trisomic mice, *n*=9 euploid littermates); (3) Dp(16)1/Yey strain (*n*=11 trisomic mice, *n*=10 euploid littermates). (L) Number of OLIG2^+^ cells per 100 µm^3^ of MGE in Ts1Cje, Ts65Dn and Dp(16)1/Yey embryos and their respective euploid littermates. Only Ts65Dn mice show a marked increase in OLIG2^+^ cells compared with euploid littermates. Mice used: (1) Ts1Cje strain (*n*=6 trisomic mice, *n*=6 euploid littermates); (2) Ts65Dn strain (*n*=9 trisomic mice, *n*=9 euploid littermates); (3) Dp(16)1/Yey strain (*n*=11 trisomic mice, *n*=10 euploid littermates). (M) Representative image showing phosphorylated histone 3 (pH3) (green) staining in the MGE of the ventral germinal zone at E15.5. Cell nuclei are stained with DAPI (blue). (N) Number of pH3^+^ cells in the MGE of Ts1Cje, Ts65Dn and Dp(16)1/Yey embryos and their respective euploid littermates. Only Ts65Dn mice show a significant increase in pH3^+^ cells compared with euploid littermates. (O-Q) Distribution of pH3^+^ into 20-µm bins starting at the ventricular surface. (O) Ts1Cje mice show a decrease only in one bin at 160 µm from the ventricular surface compared with euploid littermates. (P) Ts65Dn show a consistent increase in the area corresponding to the SVZ of the MGE (bins 140-260 µm from the ventricular surface) compared with euploid littermates. (Q) Dp(16)1/Yey shows no change in pH3^+^ cells by bin compared with euploid littermates. Mice used in N-Q: (1) Ts1Cje strain (*n*=6 trisomic mice, *n*=6 euploid littermates); (2) Ts65Dn strain (*n*=9 trisomic mice, *n*=9 euploid littermates); (3) Dp(16)1/Yey strain (*n*=11 trisomic mice, *n*=10 euploid littermates).

Growth abnormalities have been widely reported in fetuses and infants with DS during gestation and upon birth. These abnormalities extend to brain growth, which can be detected *in utero*. In gross analyses, only Ts65Dn embryos showed stunted somatic growth (97.24%±0.72 of euploid; *P*<0.01), while Dp(16)1/Yey embryos showed a nonsignificant increase in body size (110.17%±5.07 of euploid; n.s.) and Ts1Cje embryos showed no changes (Fig. 2A,B). Ts65Dn embryos were also the sole model that exhibited a decrease in rostrocaudal brain length (97.39%±1.38 of euploid; *P*<0.05) but showed no difference in mediolateral brain length (Fig. 2C,D). Neither Ts1Cje nor Dp(16)1/Yey embryos had any measurable gross brain defects at E15.5 (Fig. 2C,D).

In experiments in which Ts1Cje and Dp(16)1/Yey mice were bred onto a hybrid background matching that of Ts65Dn animals, no changes were again observed in crown-rump, or mediolateral/rostrocaudal brain lengths in Ts1Cje (trisomy passed through the paternal line) or Dp(16)1/Yey mice (trisomy passed through the maternal line; identical to Ts65Dn breeding) compared with their euploid littermates (Fig. S2A,B). These results suggest that neither the presence of a hybrid background nor maternal trisomy [tested here in Dp(16)1/Yey] influence the prenatal somatic and brain growth phenotypes in these mouse models.

#### Expansion of the dorsal pallium during embryonic neurogenesis

Measurements of the dorsal telencephalic germinal zone thickness demonstrated that Ts1Cje mice had no significant changes in the ventricular/subventricular zones (VZ/SVZ), subplate/cortical plate (SP/CP), or the overall pallial expansion compared with euploid littermates (Fig. 2E,F). However, there was a significant increase in the thickness of the intermediate zone (IZ) (121.53%±5.34 of euploid; *P*<0.05) (Fig. 2F). Ts65Dn embryos showed no change in VZ/SVZ thickness, but showed a significant decrease in IZ thickness (87.00%±1.46 of euploid; *P*<0.05) and overall pallial thickness (92.71%±1.69 of euploid; *P*<0.05) along with a trend towards a decrease in SP/CP thickness (88.31%±1.92 of euploid; *P*=0.10) (Fig. 2F). Dp(16)1/Yey mice showed no significant changes in any layer of the dorsal germinal zone or in the overall dorsal pallium (Fig. 2F).

#### Quantification of neurogenesis and neurogenic output in the dorsal and ventral telencephalic germinal zones

In previous studies, we assessed neurogenesis in Ts65Dn embryos at E14.5 using 5-bromo-2′-deoxyuridine (BrdU) pulse labeling. We showed that the number of BrdU-labeled cells was decreased in the dorsal telencephalon, but increased in the ventral telencephalon (Chakrabarti et al., 2010, 2007). However, these abnormalities in neurogenesis were not observed in Dp(16)1/Yey mice (Goodliffe et al., 2016). Here, we assessed all three models side by side at a different gestational age (E15.5) with a different *in vivo* neurogenesis assay using 5-ethynyl-2′-deoxyuridine (EdU). We quantified progenitor numbers to determine whether neurogenesis in either germinal zone is different in trisomic mice compared with their respective euploid littermates (Fig. 2G-Q).

Our data confirm that there is a significant reduction in neocortical neurogenesis in Ts65Dn forebrains. Specifically, we found a decrease in the percent of EdU-labeled cells in the VZ/SVZ of the dorsal telencephalon (88.48%±0.44 of euploid; *P*<0.01). No change was observed in Ts1Cje or Dp(16)1/Yey forebrains (Fig. 2G-J).

We also measured mitosis and progenitor cell number and distribution within the medial ganglionic eminence (MGE) of the ventral germinal zone, the birthplace of oligodendrocytes (OLs) and tangentially migrating cortical interneurons (INs) (Fig. 2K-Q). In Ts65Dn MGE, phosphorylated histone 3 (pH3) staining showed an increase in mitotically active progenitors in the SVZ (137.28%±8.76 of euploid; *P*<0.05) (Fig. 2M,N; previously reported in Chakrabarti et al., 2010). To characterize the types of progenitors within this region, we used an oligodendrocyte transcription factor 2 (OLIG2) antibody to specifically mark OL and IN progenitors (Lu et al., 2000; Miyoshi et al., 2007; Takebayashi et al., 2000). The number of OLIG2^+^ cells was increased in Ts65Dn animals (Fig. 2L; previously reported in Chakrabarti et al., 2010). In contrast, no significant changes in pH3 labeling or OLIG2 labeling were found in Ts1Cje MGE (except a decrease in pH3 staining in one abventricular bin at 160 μm) (Fig. 2K-Q) or Dp(16)1/Yey MGE (Fig. 2K-Q; data previously shown in Goodliffe et al., 2016).

### Neonatal behavior

**Fig. 3.**
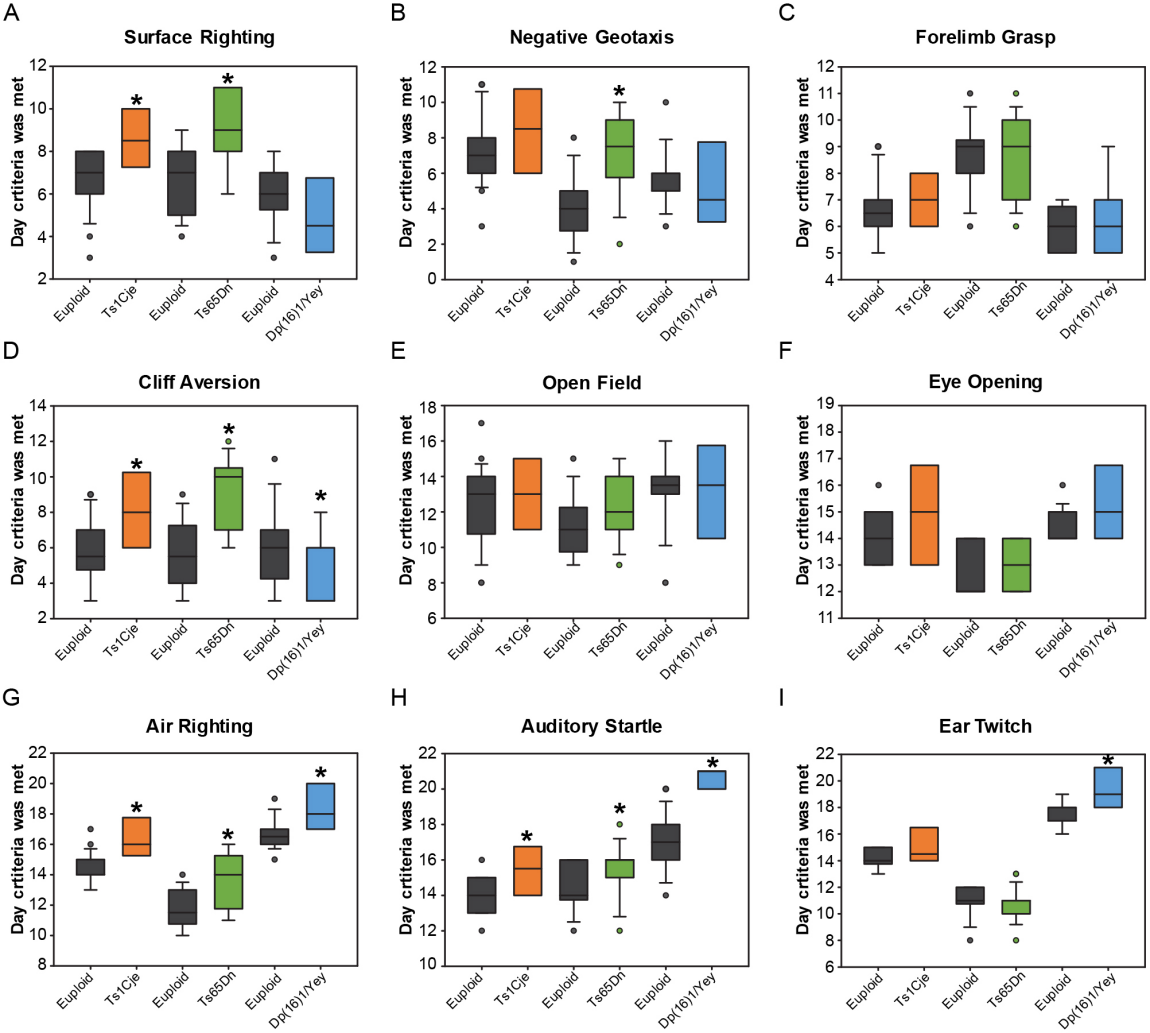
**Developmental milestones in male Ts1Cje, Ts65Dn and Dp(16)1/Yey neonates.** Developmental milestones were measured on a daily basis between birth and P21 in Ts1Cje mice (*n*=32 trisomic mice, *n*=64 euploid littermates); Ts65Dn mice (*n*=34 trisomic mice, *n*=23 euploid littermates); and Dp(16)1/Yey mice (*n*=30 trisomic mice, *n*=72 euploid littermates). Graphs showing day on which criteria were met on each task in trisomic mice compared with euploid littermates. Plots show median value for each group tested, first and third quartiles, data range and outliers; *P<0.05. (A) On surface righting, only Ts1Cje and Ts65Dn mice show an impairment compared with their euploid littermates. (B) On negative geotaxis, only Ts65Dn mice show a marked impairment compared with their euploid littermates. (C) On forelimb grasp, all trisomic mice perform similarly to their euploid littermates. (D) On cliff aversion, Ts1Cje and Ts65Dn mice show a significant impairment, whereas Dp(16)1/Yey mice show an improvement, compared with their respective euploid littermates. (E) On open field, all trisomic mice perform similarly to their euploid littermates. (F) On eye opening, all trisomic mice perform similarly to their euploid littermates, showing that there was no confound during testing from lack of vision in trisomic mice. (G) On air righting, all trisomic mice show an impairment compared with their euploid littermates. (H) On auditory startle, all trisomic mice show an impairment compared with their euploid littermates. (I) On ear twitch, only Dp(16)1/Yey mice show an impairment compared with euploid littermates.

Newborns with DS exhibit hypotonia and delays in achieving developmental milestones (Horovitz and Matson, 2011). Using the experimental paradigm established in Olmos-Serrano et al., 2016b we investigated early postnatal development in Ts1Cje, Ts65Dn and Dp(16)1/Yey mice along with their euploid littermates from birth until weaning (P21) (Fig. 3; Figs S3-S6). Despite the mild abnormalities in embryonic corticogenesis in Ts1Cje mice, and lack thereof in Dp(16)1/Yey mice, analysis of neonatal behavior was conducted to identify any cognitive and behavioral deficits and to pinpoint their timing of onset. Therefore, body weight and length, as well as motor strength, coordination and acquisition of neurological reflexes were analyzed in a single combined cohort for each of the mouse strains [combined data previously reported for Ts65Dn in Olmos-Serrano et al., 2016b and for Dp(16)1/Yey in Goodliffe et al., 2016]. Subsequently, male and female behavioral performances were independently analyzed. Overall, while the findings show that trisomic mice eventually meet criteria for each developmental milestone, each model has a unique pattern of delays.

#### Ts1Cje: both sexes combined

Combined analysis of both sexes showed that weight and body lengths were significantly decreased in Ts1Cje pups compared with their euploid littermates (Fig. S3A,B). When the percentage of mice meeting criteria for each task was analyzed daily, data showed that Ts1Cje mice performed worse on surface righting, negative geotaxis, cliff aversion, ear twitch response, air righting and auditory startle (Fig. S3). Also, fewer Ts1Cje pups opened their eyes on P14-16 compared with their euploid littermates (Fig. S3I). Ts1Cje pups performed similarly to euploid pups on the forelimb grasp and open field tasks (Fig. S3F,G). Overall, Ts1Cje mice performed significantly worse on both early and late acquired tasks (Fig. S3).

#### Ts1Cje: males versus females

When assessed separately, both Ts1Cje males and females performed similarly and experienced delays mostly in achieving late acquired tasks (Fig. 3; Fig. S6). Ts1Cje males had significantly delayed acquisition of surface righting, cliff aversion, air righting and auditory startle responses compared with euploid males (Fig. 3; Fig. S6A,D,G,H). Females had significantly delayed acquisition of negative geotaxis, cliff aversion, air righting, auditory startle and ear twitch responses compared with euploid females (Fig. S6B′,D′,G′,H′,I′).

#### Ts65Dn: both sexes combined

Weight was significantly decreased in Ts65Dn pups compared with euploid littermates (Fig. S4A). When the percent of mice meeting criteria for each task was analyzed daily, data showed that Ts65Dn pups performed worse on surface righting, negative geotaxis, cliff aversion, open field and air righting tasks compared with euploid littermates (Fig. S4). Ts65Dn performed similarly to euploid pups on the forelimb grasp, ear twitch, eye opening and auditory startle responses (Fig. S4). Similar to Ts1Cje mice, Ts65Dn mice performed significantly worse on both early and late acquired tasks (Fig. S4).

#### Ts65Dn: males versus females

Ts65Dn males performed considerably worse than Ts65Dn females (Fig. 3; Fig. S6). While Ts65Dn females showed a significantly delayed acquisition of the cliff aversion response only (Fig. S6D′), Ts65Dn males showed a significant delay in achieving surface righting, negative geotaxis, cliff aversion, air righting and auditory startle responses (Fig. 3; Fig. S6A,B,D,G,H). Thus, Ts65Dn males showed significant impairment in achieving both early and late acquired tasks, but Ts65Dn females were minimally affected (Fig. 3; Fig. S6).

#### Dp(16)1/Yey: both sexes combined

Analysis of both sexes showed that weight and body length were significantly decreased in Dp(16)1/Yey pups compared with euploid littermates (Fig. S5A,B). When the percentage of mice meeting criteria for each task was analyzed daily, data showed that Dp(16)1/Yey pups performed worse on ear twitch, air righting and auditory startle responses (Fig. S5). Also, fewer Dp(16)1/Yey pups opened their eyes on P15-16 compared with their euploid littermates (Fig. S5I). Dp(16)1/Yey pups performed similarly to euploid pups on the surface righting, negative geotaxis, forelimb grasp and open field tasks (Fig. S5). Surprisingly, a significantly larger proportion of Dp(16)1/Yey mice achieved criteria earlier in the cliff aversion task compared with euploid littermates (Fig. S5E). Overall, Dp(16)1/Yey mice performed significantly worse only on late acquired tasks (Fig. S5).

#### Dp(16)1/Yey: males versus females

Both Dp(16)1/Yey males and females performed similarly and experienced delays in achieving late acquired tasks (Fig. 3; Fig. S6). Dp(16)1/Yey males had significantly delayed acquisition of air righting, auditory startle and ear twitch responses compared with euploid males (Fig. 3; Fig. S6G,H,I). However, Dp(16)1/Yey males achieved criteria earlier for the cliff aversion task (Fig. 3D; Fig. S6D). Females had significantly delayed acquisition of negative geotaxis, air righting, auditory startle and ear twitch responses compared with euploid females (Fig. S6B′,G′,H′,I′). Dp(16)1/Yey females also had delayed eye opening compared with euploid mice (Fig. S6F′).

### Excitatory and inhibitory neuronal density

#### Postnatal defects in IN populations

**Fig. 4.**
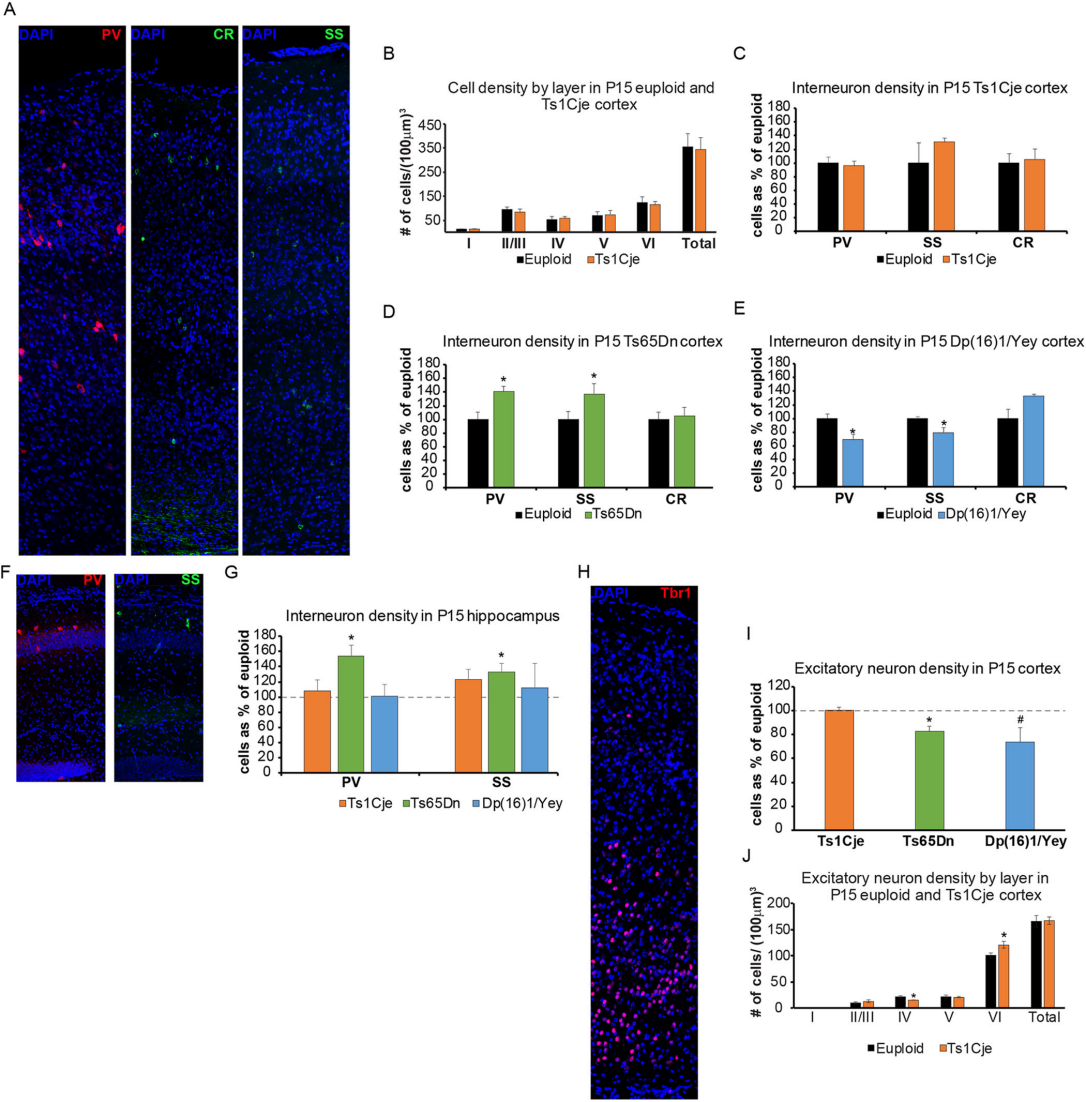
**Neuronal populations in P15 Ts1Cje, Ts65Dn and Dp(16)1/Yey forebrains.** Both excitatory and inhibitory neuronal populations were measured in the somatosensory cortices of the Ts1Cje (*n*=6 trisomic mice, *n*=6 euploid littermates), Ts65Dn (*n*=4 trisomic mice, *n*=4 euploid littermates) and Dp(16)1/Yey (*n*=4 trisomic mice, *n*=5 euploid littermates) mouse models at P15. (A) Representative images of parvalbumin (PV, red), calretinin (CR, green) and somatostatin (SS, green) inhibitory interneuron (IN) staining in the somatosensory cortex. All nuclei are counterstained with DAPI (blue). (B) Cell density by neocortical layer in P15 Ts1Cje mice compared with euploids. No change is observed in density or layer thickness (data not shown). Data are mean±s.e.m. (C-E) IN density as a percentage of total cells. Each subtype is represented separately. No change in overall density or density by neocortical layer (data not shown) is seen in Ts1Cje mice compared with their euploid littermates (C). An increase in PV^+^ and SS^+^ IN density is seen in the neocortex of Ts65Dn mice compared with their euploid littermates. No change is observed in CR^+^ INs (D). A decrease in PV^+^ and SS^+^ IN density is seen in the neocortex of Dp(16)1/Yey mice compared with their euploid littermates. No change is observed in CR^+^ INs (E). (F) Representative images of PV^+^ (red) and SS^+^ (green) INs in the dorsal hippocampus. All nuclei are counterstained with DAPI (blue). (G) No change in IN populations in the hippocampus is seen in Ts1Cje (orange bars) and Dp(16)1/Yey (blue bars) mice compared with their euploid littermates. Ts65Dn mice show an increase in both PV^+^ and SS^+^ INs in the hippocampus compared with their euploid littermates. Data are mean±s.e.m., *P<0.05. (H) Representative images of Tbr1 (red) excitatory neuron staining in the somatosensory cortex. All nuclei are counterstained with DAPI (blue). (I) Ts65Dn mice show a significant decrease in excitatory neuron numbers in the somatosensory cortex compared with their euploid littermates (green bar). Dp(16)1/Yey mice show a trend towards a decrease in excitatory neuron numbers in the somatosensory cortex compared with their euploid littermates (blue bar). Ts1Cje mice show no change in overall number of excitatory neurons in the somatosensory cortex compared with their euploid littermates. (J) However, a shift in distribution from Layer IV, favoring Layer VI, is observed in these mice. Data are mean±s.e.m., *P<0.05; ^#^*P*=0.07.

To assess the possible underlying etiology of developmental milestone abnormalities, we examined cortical and hippocampal neuronal populations to determine whether they are perturbed at a time when all three models show behavioral deficits (Fig. 4). All nuclei, counterstained with either 4′,6-diamidino-2-phenylindole, dihydrochloride (DAPI) or TO-PRO^®^-3, were counted throughout a consistent region of interest within the somatosensory cortex at P15. The total number of cells, as well as the number of cells within each neocortical layer, did not differ in Ts1Cje P15 somatosensory cortex (Fig. 4A,B). We then used somatostatin (SS), parvalbumin (PV) and calretinin (CR) to label IN subtypes and found no differences in Ts1Cje somatosensory cortex, both when overall IN numbers were counted (Fig. 4A,C) and when IN laminar position and cell density were calculated (data not shown). In contrast, both PV^+^ and SS^+^ INs were significantly increased in Ts65Dn brains in individual neocortical layers as well as overall (Fig. 4A,D; layer data in Chakrabarti et al., 2010). However, the overall number of CR^+^ cells was unchanged in Ts65Dn (Fig. 4A,D). In Dp(16)1/Yey animals, PV^+^ and SS^+^ cell numbers were decreased but CR^+^ cell numbers trended towards an increase (Fig. 4A,E; *P*=0.07; data shown in Goodliffe et al., 2016). In the hippocampus, which is also populated by MGE-derived INs, only the Ts65Dn mice showed an increase in PV^+^ and SS^+^ cell numbers (Fig. 4F,G; data shown in Chakrabarti et al., 2010); no change was observed in Ts1Cje or Dp(16)1/Yey mice [Fig. 4F,G; Dp(16)1/Yey data previously shown in Goodliffe et al., 2016].

#### Postnatal defects in excitatory neuron populations

Excitatory neurons positive for T-box brain 1 (TBR1) staining were significantly decreased in Ts65Dn cortex (Fig. 4H,I; data from Chakrabarti et al., 2010) and trended towards a decrease in Dp(16)1/Yey cortex (Fig. 4H,I; *P*=0.07; data from Goodliffe et al., 2016). In contrast, the overall number of excitatory neurons in Ts1Cje cortex was unchanged (Fig. 4H,I). Upon closer examination, we observed a misallocation of excitatory cells between neocortical layers IV and VI, leading to an increased cell number within layer VI but a decrease in layer IV in Ts1Cje brains (Fig. 4J).

### Adult behavior

Learning, memory and motor deficits are fully penetrant in people with DS and constitute major aspects of the associated intellectual disability. Because these phenotypes are present throughout the lifespan of individuals with DS, in addition to the developmental milestone assessments, we sought to test similar aspects of behavior in adult Ts1Cje, Ts65Dn and Dp(16)1/Yey mice. To do so, we utilized a battery of tests including SHIRPA, open field (OF), Morris water maze (MWM), contextual fear conditioning (CFC) and rotarod. These tests allowed us to specifically assess hippocampal-dependent spatial and contextual memory, and motor-based functions, such as locomotion, motor reflexes and motor coordination. All adult behavioral data were newly generated, except for MWM results in the Ts65Dn and Dp(16)1/Yey mice (previously published in Olmos-Serrano et al., 2016b and Goodliffe et al., 2016).

#### Reflexive behavior: SHIRPA test

Examination of over 40 different basic reflexes using the SHIRPA primary screen protocol did not reveal widespread deficits in Ts1Cje, Ts65Dn or Dp(16)1/Yey animals compared with euploid controls. All test results are summarized in Table S11.

#### Exploratory behavior and spontaneous locomotor activity: open field test

**Fig. 5.**
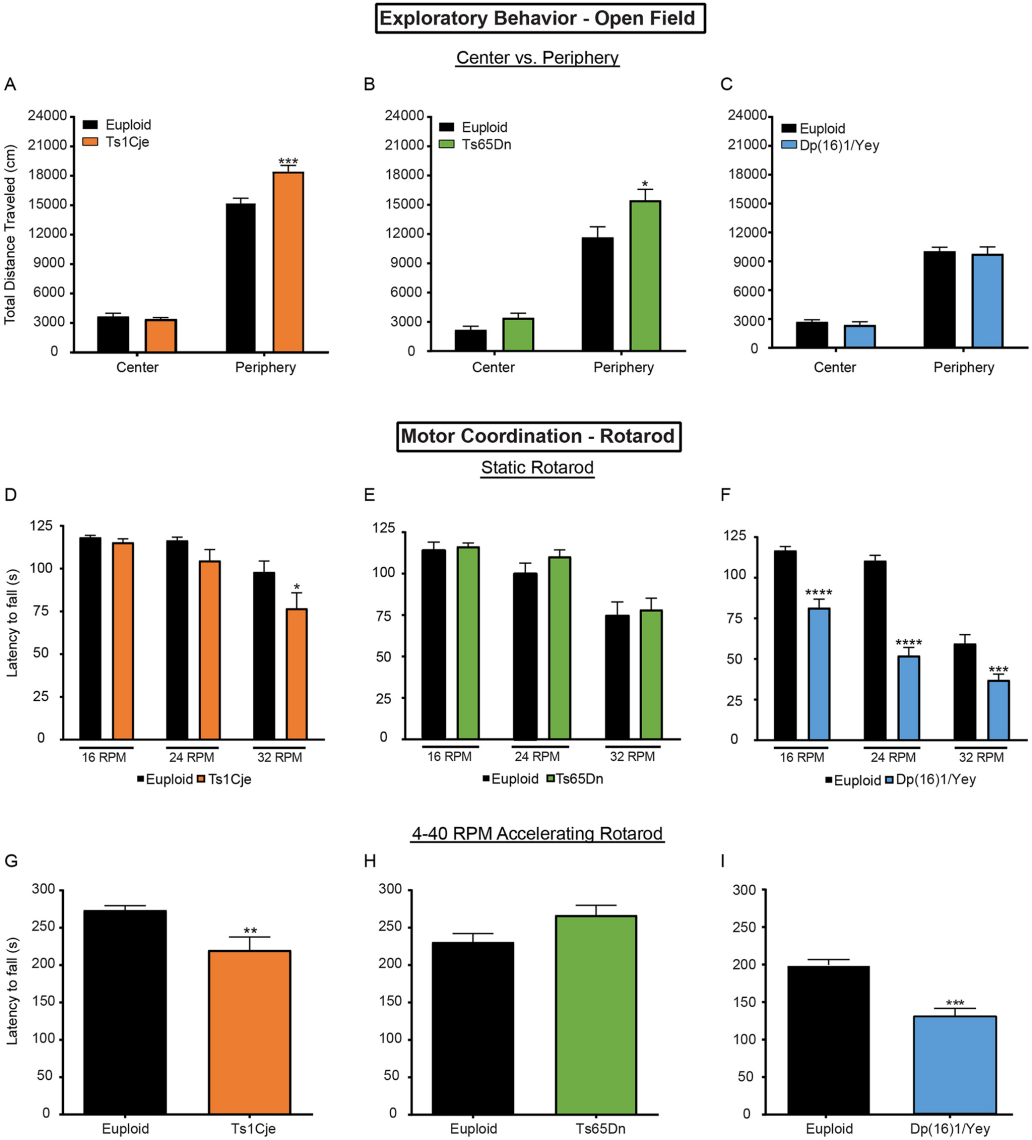
**Motor-based tasks in adult Ts1Cje, Ts65Dn and Dp(16)1/Yey males.** Exploratory motor behavior and coordination were investigated in the open field and rotarod tests in Ts1Cje mice (*n*=13 trisomic mice, *n*=15 euploid mice); Ts65Dn mice (*n*=12 trisomic mice, euploid mice=12); Dp(16)1/Yey mice(*n*=18 trisomic mice, *n*=17 euploid mice). (A-C) Measurement of distance traveled in the center versus periphery of testing space during the open field task. This measurement is a representation of exploratory behavior in animals. Ts1Cje mice travel more distance in the periphery compared with their euploid controls. Travel in the center is similar between genotypes (A). Ts65Dn mice also travel more distance in the periphery compared with their euploid controls. Travel in the center is similar between genotypes (B). Dp(16)1/Yey mice show no change in distance traveled in both center and periphery compared with their euploid controls (C). (D-F) Latency to fall during the nonaccelerating rotarod at three different speeds: 16, 24 and 32 RPM. This task measures motor coordination in animals. Ts1Cje mice only show a deficit at the highest rotational speed of 32 RPM (D). Ts65Dn mice show no difference in rotarod performance compared with a pooled cohort of B6C3Sn hybrid euploids at any speed (E). Dp(16)1/Yey mice show a marked impairment in rotarod performance at every speed compared with their euploid controls (F). (G-I) Latency to fall during the accelerating rotarod task, which gradually increases in rotational speed from 4 RPM to 40 RPM. This task measures motor coordination in animals. Ts1Cje mice show significant impairment in accelerating rotarod task compared with their euploid controls (G). Ts65Dn mice show no difference in rotarod performance compared with a pooled cohort of B6C3Sn hybrid euploids (H). Dp(16)1/Yey mice show a marked impairment in rotarod performance compared with their euploid controls (I). Data are mean±s.d., *P<0.05, **P<0.01, ***P<0.001, ****P<0.0001.

When exploratory behavior was analyzed over a 60-min open field trial period, the total distance traveled by Ts65Dn mice was significantly higher than that traveled by their euploid controls (*P*<0.05, Mann–Whitney test) (Fig. S7B), but was unchanged in Ts1Cje and Dp(16)1/Yey mice (Fig. S7A,C). Upon closer inspection, we found that in both Ts65Dn and Ts1Cje mice, the total distance traveled in the periphery, but not in the center, was significantly higher than that traveled by euploid controls (*P*<0.05 and *P*<0.001, respectively, Mann–Whitney test) (Fig. 5A,B). In contrast, the distance traveled in the center versus periphery was unchanged in the Dp(16)1/Yey mice compared with euploid mice (Fig. 5C).

Ts1Cje mice on the B6C3Sn background showed no impairments in total distance traveled compared with their euploid littermates (Fig. S2C).

Further analysis using 20-min time bins showed that both Ts65Dn and Ts1Cje mice traveled a significantly longer distance overall and in the periphery compared with their euploid controls during each 20-min period (*P*<0.05, Kruskal–Wallis test) (Fig. S7). However, Dp(16)1/Yey mice once again showed no change compared with euploid mice at any time interval (Fig. S7). Raw data are presented in Table S12.

#### Motor coordination: rotarod

In the static speed [16, 24 and 32 revolutions per minute (RPM)] test (day 1), Ts1Cje mice fell significantly faster than euploid littermates only at the highest rotational speed of 32 RPM (*P*<0.05, Mann–Whitney test) (Fig. 5D). On the other hand, Ts65Dn mice showed no differences in latency to fall compared with their euploid controls at 16, 24 and 32 RPM (Fig. 5E). Dp(16)1/Yey mice fell significantly faster than their euploid controls at all speeds tested (*P*<0.001, Mann–Whitney test) (Fig. 5F). In the accelerating speed test (day 2), both Dp(16)1/Yey and Ts1Cje mice fell significantly faster compared with their respective euploid controls, with the Dp(16)1/Yey mice showing the most severe deficits (*P*<0.001, Mann–Whitney test) (Fig. 5G,I). Similar to our findings in the static speed test, Ts65Dn mice showed no change compared with their euploid controls in the accelerating speed test (Fig. 5H). Raw data are presented in Table S12.

Ts1Cje mice on the B6C3Sn hybrid background showed no impairments compared with euploid littermates in both the static and accelerating rotarod tests (Fig. S2E,F).

#### Hippocampal-dependent contextual memory: contextual fear conditioning test

**Fig. 6.**
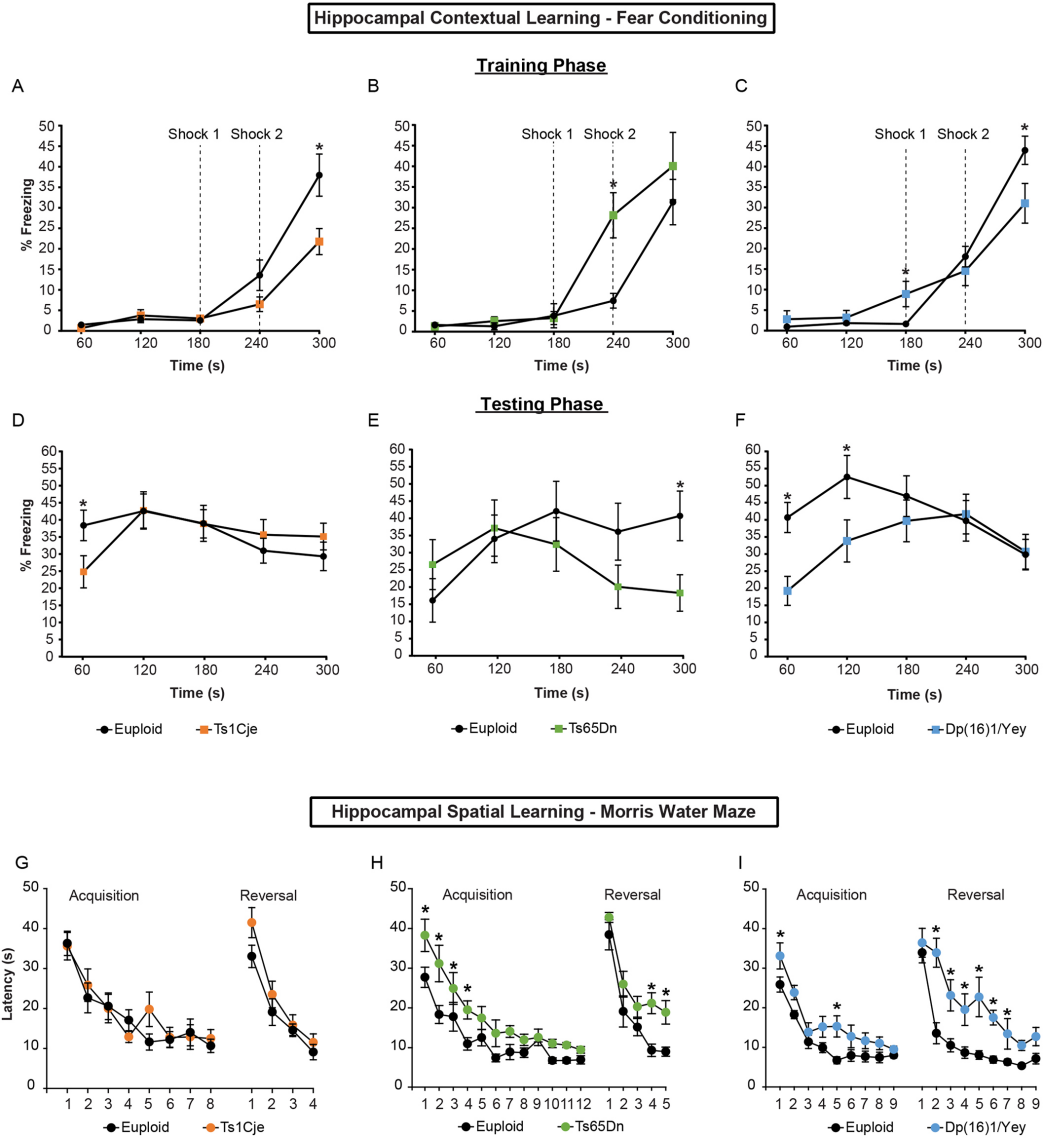
**Hippocampal-based tasks in adult Ts1Cje, Ts65Dn and Dp(16)1/Yey males.** Hippocampal-dependent spatial and contextual memory were investigated using the fear conditioning and Morris water maze (MWM) tests. (A-F) The contextual fear conditioning test has two phases: training and testing. During the training phase, mice are given two mild shocks 60 s apart. On the following day, mice are placed in the same chamber but no shocks are applied. Freezing behavior is documented. Animals used in A-F: Ts1Cje mice (*n*=13 trisomic mice, *n*=15 euploid mice); Ts65Dn (*n*=12 trisomic mice, *n*=12 euploid mice); and Dp(16)1/Yey mice (*n*=18 trisomic mice, *n*=17 euploid littermates). (G-I) The MWM test has two phases: acquisition and reversal. Both tests utilize a hidden platform to analyze learning (acquisition phase) and reversal learning (reversal phase). Mice are initially tested using a visible platform to exclude any confounds related to testing procedures or non-learning based deficits in the mice. Ts1Cje males show no deficits during either the acquisition phase or the reversal learning phase (G). Ts65Dn males show no deficits during the acquisition period after the 4 days needed to stop thigmotaxic behavior and acclimate to the task (previously published in Olmos-Serrano et al., 2016b). However, these mice show a deficit in reversal learning compared with their euploid controls (H). Dp(16)1/Yey males show impaired learning on days 1 and 5 of the acquisition phase. Additionally, these males also show a strong deficit in reversal learning compared with their euploid controls (I). Animals used in G-I: Ts1Cje mice (*n*=13 trisomic mice, *n*=11 euploid mice); Ts65Dn (*n*=14 trisomic mice, *n*=14 euploid mice); and Dp(16)1/Yey mice (*n*=13 trisomic mice, *n*=11 euploid littermates). Data are mean±s.d., *P<0.05.

During a 5-min training session on day 1, Ts1Cje mice showed less freezing behavior (21.76±3.17%) compared with euploid mice (37.95±5.13%; *P*<0.01, Mann–Whitney test) only at 300 s, otherwise these trisomic mice performed similarly to their euploid controls (Fig. 6A). In contrast, Ts65Dn mice displayed higher freezing behavior (28.15±5.47%) compared with euploid mice (7.48±1.78%; *P*<0.05, Mann–Whitney test) only at 240 s (Fig. 6B). Dp(16)1/Yey mice showed higher freezing behavior (8.91±3.10%) compared with their euploid littermates (1.63±0.65%; *P*<0.05; Mann–Whitney test) before receiving the first shock and 60 s after receiving the second shock (Fig. 6C). However, the percentage freezing was similar between the genotypes between the first and second shocks (Fig. 6C).

On testing day (day 2), Ts1Cje mice showed significantly less freezing behavior (24.80±4.75%) compared with their euploid controls (38.50±4.50%) during the first minute of testing (*P*<0.05, Mann–Whitney test) (Fig. 6D). However, Ts65Dn mice showed less freezing behavior compared with euploid littermates starting at 180 s, reaching statistical significance (Ts65Dn mice=18.27±5.31%, euploid controls=40.70±7.21%; *P*=0.016, Mann–Whitney test) only in the last minute of testing (Fig. 6E). Dp(16)1/Yey mice showed significantly less freezing behavior (19.24±4.24%) compared with euploids (40.70±4.42%; *P*<0.01, Mann–Whitney test) between 0 s and 60 s (Fig. 6F) and between 60 s and 120 s [Dp(16)1/Yey: 33.83±6.16%; euploids: 52.54±6.29%; *P*<0.05] (Fig. 6F). Raw data are presented in Table S12.

In experiments in which Ts1Cje mice were bred onto a B6C3Sn hybrid background, there were no impairments compared with euploid littermates on any aspects of the contextual fear conditioning task (Fig. S2D).

#### Hippocampal-dependent spatial memory: MWM test

##### Ts1Cje

We first employed a cued learning protocol to ensure that mice had the ability to learn to swim to a visual goal. Both groups significantly decreased their latency to find the visible platform over 4 days (*P*<0.001, data not shown). We did not find significant differences between groups (*P*=0.226, data not shown), indicating that both groups were able to learn the basic skill of swimming towards a visible goal and climbing onto the platform before being rescued. Analysis of time spent in the periphery during visible platform training revealed no significant difference between groups (data not shown). We concluded that cued learning ability was similar between genotypes, ruling out procedural deficits.

The day after the visual test ended, both genotypes were tested for their ability to learn the location of a hidden platform. Overall, as expected, both genotypes improved their performance over successive trial days as measured by decreased latencies (*P*<0.001; euploid=13, Ts1Cje=11; Fig. 6G) and swimming distance (data not shown). We did not find significant differences between genotypes in latency [*F*_(1,154)_=0.155, *P*=0.697; euploid=13, Ts1Cje=11; Fig. 6G], swimming distance and speed (data not shown). We also did not find significant differences in thigmotaxis, i.e. time spent in the periphery of the tank (data not shown). When reversal learning was tested, both genotypes also improved their performance over successive trial days as measured by decreased latencies (*P*<0.001; euploid=13, Ts1Cje=11; Fig. 6G) and swimming distance (data not shown). Surprisingly, we did not find significant differences between genotypes in latency [*F*_(1,66)_=2.693, *P*=0.115; euploid=13, Ts1Cje=11; Fig. 6G], swimming distance and speed (data not shown).

We also tested the reference memory the day after the acquisition and reversal period by removing the platform and allowing mice to swim freely for 60 s. Both probe trials revealed a selective quadrant search, indicating proper memory consolidation of the platform location, and no differences between genotypes were found [*F*_(1,66)_=2.38, *P*=0.991 for probe trial and *F*_(1,266)_=1.08, *P*=0.773 for probe trial after reversal; euploid=13, Ts1Cje=11; Fig. S8A-C]. We also tested the reference memory 3 days after the last day of the reversal learning period and also did not find any significant differences between genotypes. Similarly, we did not find significant differences between genotypes in proximity and number of virtual platform crossings during the probe trial (data not shown). Overall, these results show that Ts1Cje mice do not exhibit learning and memory deficits using this behavioral paradigm.

##### Ts65Dn

In the cued learning protocol, both groups significantly decreased their latency to find the visible platform over 4 days [*F*_(3,81)_=119.414, *P*<0.001; data not shown]. We did not find significant differences between groups [*F*_(1,81)_=1.182, *P*=0.287], indicating that both groups were able to learn the basic skills of swimming towards a visible goal and climbing onto the platform before being rescued. Euploid mice showed longer swim paths [*F*_(1,81)_=6.655, *P*=0.016; data not shown] and higher swimming speeds [*F*_(1,81)_=7.628, *P*=0.010; data not shown]. Ts65Dn mice exhibited similar performances in the last 2 days compared with euploid mice. Analysis of time spent in the periphery during visible platform training revealed no significant differences between groups [*F*_(1,81)_=1.067, *P*=0.311; data not shown]. Again, we concluded that cued learning abilities were similar between genotypes, ruling out procedural deficits.

The day after the visual test ended, both genotypes were tested for their ability to learn the location of a hidden platform. Overall, as expected, both genotypes improved their performance over successive trial days, as measured by decreased latencies and swimming distance [euploid animals: *F*_(11,286)_=27.998, *P*<0.001; trisomic animals: *F*_(11,286)_=30.887, *P*<0.001; Fig. 6H]. We found significant differences between genotypes, suggesting deficits in learning in Ts65Dn mice. However, previous analysis showed that these differences were caused by thigmotaxis, and that Ts65Dn mice need 3-4 days to acclimate to the task before their underlying learning and memory capabilities can be fully measured (Olmos-Serrano et al., 2016b). Importantly, we found that Ts65Dn mice exhibit normal spatial learning and memory following this thigmotaxis period (Fig. 6H). During the reversal testing phase, we uncovered significant differences between groups in latency [*F*_(1,104)_=7.504, *P*=0.011; latencies *q*=3.874; Fig. 6H] and swimming distance (data not shown). This showed a lack of flexibility in learning in Ts65Dn mice.

During the probe trial, both euploid and Ts65Dn mice displayed a selective quadrant search demonstrating that both groups formed a cognitive map to find the platform (*P*<0.05; Fig. S8D-F). However, Ts65Dn animals spent significantly less time in the target quadrant compared with euploid mice after the probe trial and probe trial reversal periods (*P*<0.05; Fig. S8D-F). This pointed to a long-term memory deficit in Ts65Dn mice. More detailed analyses of virtual platform crossings and proximity to the virtual platform uncovered distinct behavior in Ts65Dn compared with euploid mice (Olmos-Serrano et al., 2016b). Overall, these data indicate that Ts65Dn mice have a spatial long-term memory impairment that is most accentuated during reversal periods.

##### Dp(16)1/Yey

Similar to Ts1Cje and Ts65Dn mice, Dp(16)1/Yey animals were first tested in a cued learning protocol to assess their ability to swim to a visible goal. Both genotypes learned to swim toward a submerged platform identified by a flag, significantly decreasing their latency over 4 days [*F*_(3,66)_=98.174, *P*<0.001, data not shown]. Two-way repeated-measures ANOVA revealed no significant differences between genotypes in the latency to find the cued platform [*F*_(1,66)_=0.343, *P*=0.564], distance traveled [*F*_(1,66)_=1.595, *P*=0.220] or thigmotaxis [*F*_(1,66)_=0.0994, *P*=0.755, data not shown]. Dp(16)1/Yey animals swam more slowly than euploids to the visible platform [*F*_(1,66)_=10.795, *P*=0.003, data not shown], but this did not affect their performance.

During the hidden platform testing phase, Dp(16)1/Yey and control groups learned the hidden platform location decreasing their latency and swimming distance [latency: *F*_(8,176)_=13.542, *P*<0.001; Fig. 6I; distance: *F*_(8,176)_=14.614, *P*<0.001, data not shown]. However, there was a significant difference between genotypes in these two measures [latency: *F*_(1,176)_=9327, *P*=0.006; distance: *F*_(1,176)_=4.555, *P*=0.044; Fig. 6I]. Post hoc Tukey test comparisons indicated that Dp(16)1/Yey mice performed particularly worse on days 1 and 5 in both latency and distance (*P*<0.05; Fig. 6I). There was no overall difference between genotypes in swimming speed (data not shown), and neither genotype exhibited thigmotaxic behavior (data not shown). Interestingly, the reversal phase revealed strong deficits in Dp(16)1/Yey mice in latency and swimming distance [latency: *F*_(1,176)_=55.569, *P*<0.001; Fig. 6I; distance: *F*_(1,176)_=29.364, *P*<0.001, data not shown]. Importantly, no difference was seen in swimming speed between groups [*F*_(1,176)_=2.293, *P*=0.144, data not shown].

Both probe trials revealed a selective quadrant search, indicating proper memory consolidation of the platform location [trial 1: *F*_(3,66)_=92.886, *P*<0.001; and trial 2: *F*_(3,66)_=75.616, *P*<0.001; Fig. S8G-I]. In particular, both euploid and Dp(16)1/Yey animals spent more time in the proper quadrants in the acquisition and reversal periods, respectively; *P*<0.05; Fig. S8G-I). However, Dp(16)1/Yey mice spent significantly less time than their euploid littermates in the proper quadrant during the reversal probe trial, indicating memory deficits in Dp(16)1/Yey mice. We also found significant differences between genotypes in proximity and the number of virtual platform crossings for both the initial 30 s and the entire 60 s during the probe trial in the reversal period (*P*<0.01; data not shown). Overall, these results demonstrate that, like Ts65Dn mice, Dp(16)1/Yey animals exhibit learning and memory deficits specific to memory extinction and relearning.

### Adult brain gene expression studies

#### DEX genes at different FDR stringency cut-offs

Similar to what was observed in the embryonic forebrain, Ts65Dn adult brains had the largest number of DEX genes while Dp(16)1/Yey adult brains had the lowest number of DEX genes at FDRs <5%, <10% and <20% (Table S1). Additionally, regional clustering of DEX genes within the brain differed between models, indicating spatially restricted aberrations within the mature adult trisomic brains (Tables S1 and S13-S15).

**Fig. 7.**
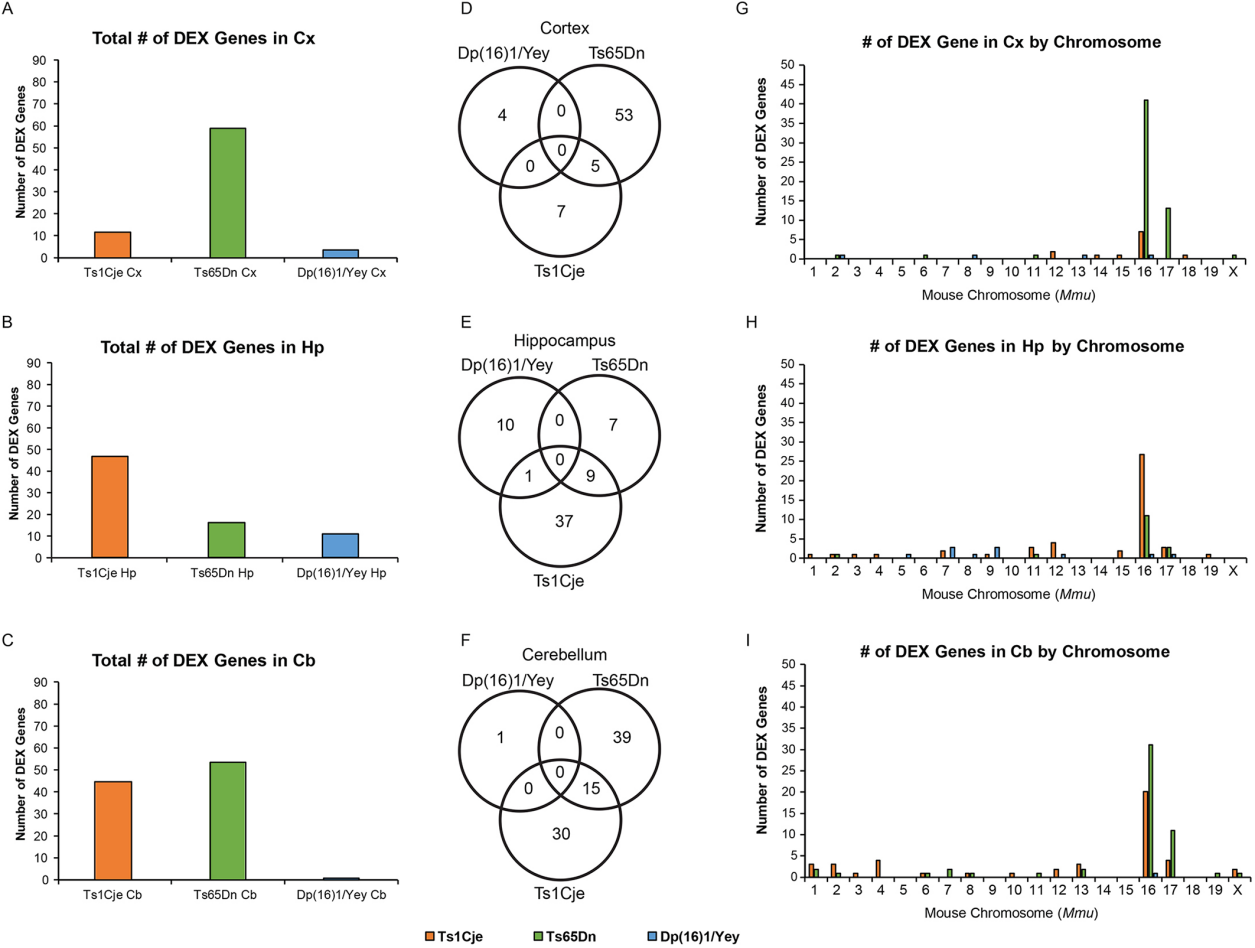
**Number of DEX genes and their chromosomal clustering in adult Ts1Cje, Ts65Dn and Dp(16)1/Yey brains by region.** Global gene expression analysis of cortex, hippocampus and cerebellum in adult male Ts1Cje mice (*n*=5 per genotype); Ts65Dn mice (*n*=5 per genotype); and Dp(16)1/Yey mice (*n*=5 per genotype). DEX genes were designated as such using a Benjamini-Hochberg FDR cut-off of <10%. (A-C) Overall number of DEX genes in each model by region. Dp(16)1/Yey mice display the lowest number of total DEX genes. Ts1Cje and Ts65Dn mice display a similar number of total DEX genes to one another, but these genes differ in identity and in chromosomal location in each model (G-I). (D-F) Venn diagrams showing the number of common DEX genes among the models by brain region. (G-I) Analysis showing genome-wide chromosomal clustering of DEX genes in Ts1Cje mice, Ts65Dn mice and Dp(16)1/Yey mice by brain region.

Once again we chose an FDR <10% to identify DEX genes for downstream analyses. At this FDR, Ts1Cje mice had more DEX genes in the hippocampus (47 DEX genes: 34 upregulated and 13 downregulated) and cerebellum (45 DEX genes: 35 upregulated and 10 downregulated) compared with cortex (12 DEX genes: nine upregulated and three downregulated) (Fig. 7A-C; Tables S13-S15). Ts65Dn mice had the largest number of DEX genes in the cortex (58 DEX genes: 55 upregulated and three downregulated), and cerebellum (54 DEX genes: 48 upregulated and six downregulated), compared with hippocampus (16 DEX genes: all upregulated) (Fig. 7A-C; Tables S13-S15). Dp(16)1/Yey mice had more DEX genes in the hippocampus (11 DEX genes: six upregulated and five downregulated) compared with cortex (four DEX genes: three upregulated and one downregulated) and cerebellum (one DEX gene: upregulated) (Fig. 7A-C; Tables S13-S15).

In cortex, Ts1Cje and Ts65Dn mice had five DEX genes in common, while in hippocampus they shared nine DEX genes, and in cerebellum they shared 15 DEX genes (Fig. 7D,H). Dp(16)1/Yey had no DEX genes in common with Ts65Dn mice in any brain region (Fig. 7D-F). Dp(16)1/Yey mice also had no DEX genes in common with Ts1Cje, except for one in the hippocampus (Fig. 7D-F). In all three brain regions, there were no DEX genes that were common to all three mouse models (Fig. 7D-F). A list of all DEX genes by region is presented in Tables S13-S15 and the number of DEX genes from each chromosome is presented by region in Fig. 7G-I. Generally, the majority of DEX genes in each brain region were clustered within the triplicated segment, but we did not observe any other chromosomal clustering throughout the genome (Fig. 7G-I).

#### Analysis of non-MMU16 aneuploid genes in the Ts65Dn and Ts1Cje models

In the Ts65Dn adult brain, several of the triplicated MMU17 centromeric genes were upregulated, leading to the following regional distribution: eight DEX genes in cortex, 13 DEX genes in hippocampus and 11 DEX genes in cerebellum (Table S16). These triplicated genes are not orthologous to any genes on HSA21.

Similarly, in the Ts1Cje adult brain several of the monosomic genes within the MMU12 telomeric region were differentially expressed. Except for *Dnah11*, which is consistently upregulated in all brain regions, *Tmem196* is the only other gene that is downregulated in cortex and hippocampus, while *Sp4* is downregulated in hippocampus and *Itgb8* is downregulated in both hippocampus and cerebellum (Table S17).

#### Comparison of genome-wide effects in Ts1Cje, Ts65Dn and Dp(16)1/Yey adult brain

**Fig. 8.**
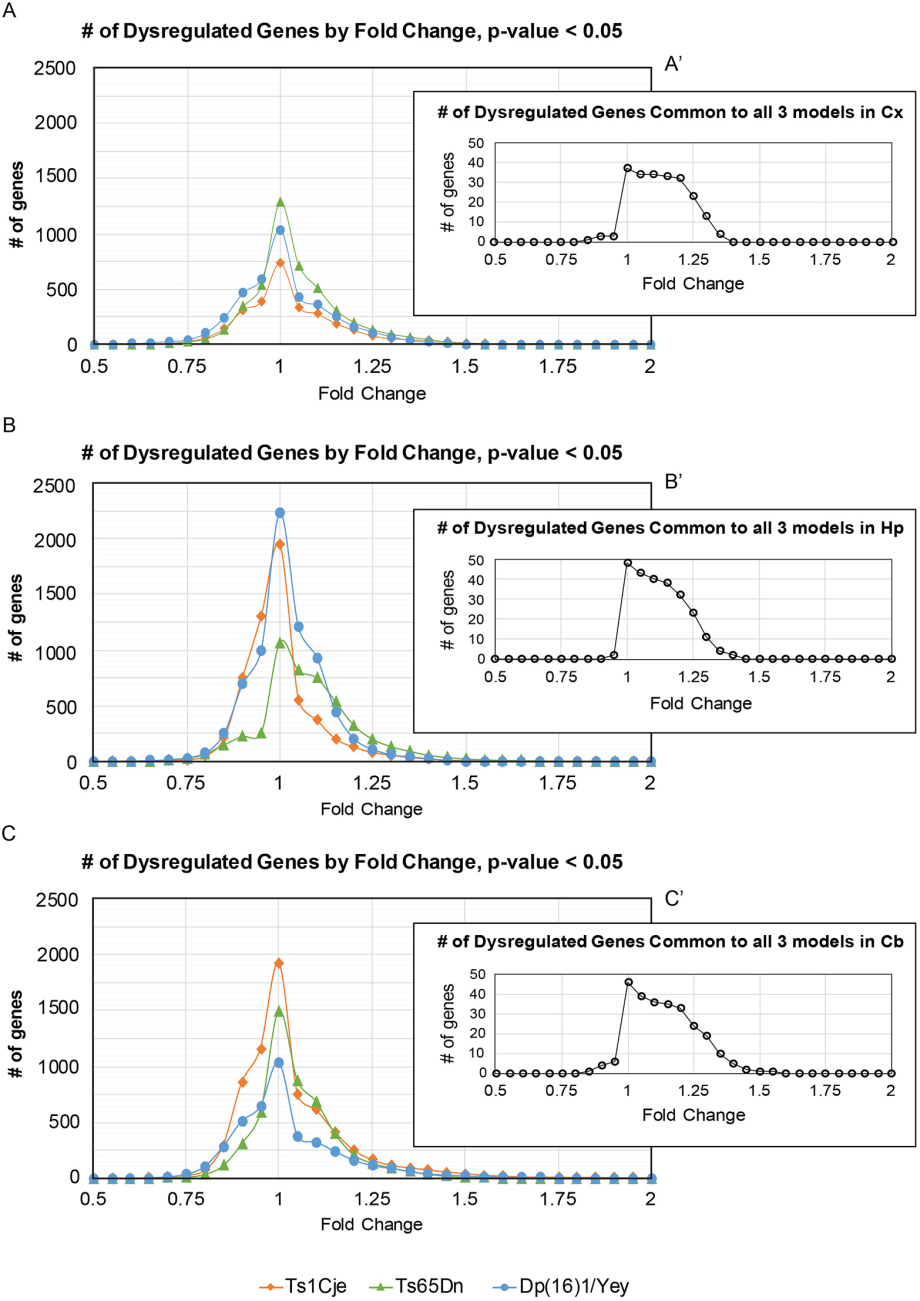
**Number of dysregulated genes by** **FC** **in adult male Ts1Cje, Ts65Dn and Dp(16)1/Yey brains by region.** (A-C) Distribution of dysregulated genes in each brain region by FC, showing that the majority of dysregulated genes have small magnitude FCs that lie between 0.75 and 1.25. These genes show a significant FC value in trisomic mice compared with their euploid controls, *P*<0.05. (A′-C′) Distribution of dysregulated genes that are common to all three models, displayed by brain region. The majority of dysregulated genes in common cluster between 0.8 and 1.3 FC.

Similar to embryonic gene expression analyses, quantifying the number of significantly altered genes (*P*<0.05) showed that the highest number of altered genes and highest number of genes in common between the models fell between the range of FC<1.25 and FC>0.75 (Fig. 8A-C). This, once again, indicated that, in adult trisomic animals, the majority of gene expression differences were small in magnitude (Fig. 8A′-C′). Therefore, as was done for the embryonic forebrain tissue, we utilized GSEA for a holistic analysis and then generated an MDG list for subsequent DAVID analysis to identify pathways and to complement the GSEA data.

Using FC<0.8 and >1.2 with a *P*-value of 0.05 as cut-off revealed that Dp(16)1/Yey cortex had the largest number of MDGs (267) compared with Ts65Dn cortex (246) and Ts1Cje cortex (189) (Table S18). However, Ts65Dn cortex showed the highest number of upregulated genes (196) compared with Ts1Cje cortex (127) and Dp(16)1/Yey cortex (164), while Dp(16)1/Yey cortex had the largest number of downregulated genes (103) compared with Ts65Dn cortex (50) and Ts1Cje cortex (62) (Table S18). There were 49 upregulated genes in common between Dp(16)1/Yey and Ts65Dn cortex, 36 between Dp(16)1/Yey and Ts1Cje cortex, and 46 between Ts65Dn and Ts1Cje cortex, with 32 genes being upregulated in the cortex of all three strains. In contrast, only four genes were downregulated in two of the three models (Table S18).

Although Ts65Dn adult brains exhibited the lowest number of DEX genes in the hippocampus, the number of MDGs within the hippocampus was much larger (319 upregulated and 69 downregulated) (Table S19). Dp(16)1/Yey hippocampus also had a large number of MDGs (202 upregulated and 75 downregulated), while Ts1Cje hippocampus showed the lowest number of MDGs (127 upregulated and 49 downregulated) (Table S19). There were 46 upregulated genes in common between Dp(16)1/Yey and Ts65Dn hippocampus, 46 between Dp(16)1/Yey and Ts1Cje hippocampus, and 41 between Ts65Dn and Ts1Cje hippocampus, with 32 genes being upregulated in the hippocampus of all three models. Eight genes were downregulated in two models (Table S19).

Finally, in the cerebellum, Ts1Cje mice had the largest number of MDGs (256 upregulated and 73 downregulated), followed by Dp(16)1/Yey mice (165 upregulated and 106 downregulated), and Ts65Dn mice (214 upregulated and 35 downregulated) (Table S20). There were 48 upregulated genes in common between Dp(16)1/Yey and Ts65Dn cerebellum, 75 between Dp(16)1/Yey and Ts1Cje cerebellum, and 48 between Ts65Dn and Ts1Cje cerebellum, with 33 genes being upregulated in the cerebellum of all three strains. Three genes were downregulated in two models (Table S20).

#### Dysregulated pathways and cellular processes

GSEA findings show that, similar to embryonic forebrains, adult Ts1Cje, Ts65Dn and Dp(16)1/Yey mice show consistent upregulation of interferon signaling and immune response pathways in all brain regions examined. Ts1Cje and Dp(16)1/Yey brains exhibited a downregulation in FGF receptor signaling, while Ts1Cje and Ts65Dn brains exhibited an upregulation in the JAK-STAT and netrin 1 signaling pathways and in Golgi complex-related gene sets. Ts65Dn and Dp(16)1/Yey brains had a downregulation of transcriptional activity and RNA polymerase I-dependent transcription, while pyruvate metabolism, cysteine-dependent peptidase activity and MHC class II antigen presentation were upregulated in these mice. No common consistently downregulated pathways were observed in all models by brain region (Table S5).

DAVID analysis (all pathways reported in Tables S21-S23 and summarized in Table S24) revealed the following:

1. In the cortex, Ts65Dn mice had more dysregulated pathways than Dp(16)1/Yey and Ts1Cje mice. Gene sets associated with G-protein signaling and olfactory transduction were largely downregulated in all three strains, whereas immunological pathways were upregulated in all three strains. Additionally, Ts65Dn mice had a distinct pathway profile involving terms related to neurogenesis and behavior.
2. In the hippocampus, Dp(16)1/Yey mice had the largest number of dysregulated pathways. Genes associated with oxidoreductase activity and endoplasmic reticulum function were upregulated in all three models, while genes associated with olfactory transduction were downregulated in Ts65Dn and Dp(16)1/Yey mice. When Ts1Cje and Dp(16)1/Yey mice were compared, gene sets associated with extracellular exosomes, mitochondrial membrane and transferase activity were commonly upregulated. Finally, Ts1Cje and Ts65Dn mice exhibited upregulation of genes involved in the oxidative stress response.
3. In the cerebellum, Dp(16)1/Yey and Ts1Cje mice had the highest number of dysregulated pathways compared with the Ts65Dn mice. As in the cerebral cortex and hippocampus, immune response and interferon signaling were highly upregulated in all three mouse models. Ts1Cje and Dp(16)1/Yey mice displayed a significant upregulation of JAK-STAT signaling, GTPase activity and double-stranded RNA binding, and downregulation of G-protein-coupled receptor signaling (i.e. olfactory receptor activity). When compared with the other two models, Ts65Dn exhibited a distinct profile of dysregulated pathways.

#### miRNA expression

miRNA expression was analyzed in Ts1Cje, Ts65Dn and Dp(16)1/Yey adult brains to assess whether miRNA-dependent regulation could be related to the low number of DEX genes observed in these animals and, in particular, in Dp(16)1/Yey mice. Ts1Cje cortex and hippocampus had one and two upregulated miRNAs, respectively (Table S25). No change in miRNA expression was seen in Ts1Cje cerebellum (Table S25). Ts65Dn mice had three marginally dysregulated miRNAs in cortex (all downregulated), four in hippocampus (three upregulated, including miR155; one downregulated), and three in cerebellum (all upregulated) (Table S25). Lastly, Dp(16)1/Yey mice had six marginally dysregulated miRNAs in cortex (five upregulated and one downregulated), three in hippocampus (one upregulated and two downregulated), and four in the cerebellum (one upregulated and three downregulated) (Table S25).

#### qRT-PCR validation of adult microarray findings

Similar to our embryonic microarray validation, we validated the adult microarray findings using the same set of genes – *Hspa13*, *App*, *Ttc3* and *Rfx5* – in the cortex and hippocampus of Ts1Cje, Ts65Dn and Dp(16)1/Yey mice. Both microarray and qRT-PCR data showed that gene expression changes were consistent across methods and with the gene dosage in each mouse model (Table S26A,B).

## DISCUSSION

This novel comparative study highlights numerous significant differences in brain development, gene expression and behavior in the Ts1Cje, Ts65Dn and Dp(16)1/Yey mouse models of DS (summarized in Table 1). The extent of variation between the different models was unexpected, because it has been widely accepted that segmental trisomy of MMU16 is a valid model for triplication of HSA21 and that triplication of the orthologous genes leads to common phenotypes (Davisson et al., 1990; Li et al., 2007; Reeves et al., 1995; Sago et al., 1998; Sérégaza et al., 2006). The various genetic, morphological and behavioral differences between strains indicate distinct etiologies, obscuring the identification of a common mechanism for DS-relevant neurological deficits across the models. Another important conclusion from these studies is that frank alterations in prenatal brain growth are not required for later postnatal or adult behavioral deficits in a DS model. Indeed, one of the models [Dp(16)1/Yey] displays abnormalities in juvenile and adult motor/cognitive tests without any appreciable prenatal brain morphogenesis deficits. These data reframe interpretation of previous reports in the literature and have important implications for future use of these models in understanding the neurobiology of DS and in developing novel therapies.

### Summary of brain phenotypes by model

#### Ts1Cje

Our transcriptome data indicate that markers of cerebral cortex development and cell proliferation, including kinetochore organization and metaphase/anaphase checkpoint regulators, are upregulated in E15.5 Ts1Cje forebrains. These gene expression data are supported by our neurogenesis experiments in which we found a ∼20% increase in the thickness of the dorsal IZ at E15.5. Previous reports, however, showed that Ts1Cje embryos had a decreased overall brain size as well as decreased cortical neurogenesis at E14.5 (Ishihara et al., 2010). Contrary to our findings, these studies also showed that Ts1Cje mice exhibited an increase in proliferation in the MGE at E14.5, followed by enlarged ventricles and decreased hippocampal proliferation postnatally (Ishihara et al., 2010, 2014). These differences in histological findings might be related to differences in methodology (Qu et al., 2011) or the fact that we analyzed a larger cohort in our current study. Additionally, these differences could have arisen from a possible phenotypic drift known to sometimes occur in fully inbred colonies (Casellas, 2011).

At P15, Ts1Cje forebrains show no change in total cell density, cortical excitatory neuron density, or cortical and hippocampal inhibitory IN densities. However, there is a shift in the laminar position of excitatory neurons, indicating some perturbation in cellular allocation in the somatosensory cortex. Interestingly, despite the lack of frank changes in pre- and perinatal brain morphology, Ts1Cje mice exhibit deficits in both early and late developmental milestones. However, these animals do not show widespread deficits in motor- and hippocampal-based tasks as adults. Importantly, we found no debilitating impact of a hybrid background strain on any prenatal or postnatal phenotypes in Ts1Cje, eliminating this factor as a potential confounding variable in our findings in Ts65Dn mice.

Gene expression data show that similar numbers of DEX genes can be found in both Ts1Cje and Ts65Dn brains during gestation and in adulthood, but there are very few DEX genes in common between both models. This lack of similarity in gene expression could explain the phenotypic differences in Ts1Cje and Ts65Dn embryos and adults. Additionally, differential expression in four of the seven distal MMU12 genes might contribute to the lack of phenotype. For example, we showed that in embryonic Ts1Cje forebrain there was a ∼25% decrease in *Tmem196* expression (*P*=0.0008). Previous work has shown that knockdown of *Tmem196* increases proliferation and inhibits apoptosis and cell cycle arrest in rat lung (Liu et al., 2015). Perhaps *Tmem196* has similar antiproliferative pro-apoptotic properties in the developing brain and its downregulation contributes to the observed increase in thickness of the dorsal pallium. Our study does not directly assess the functional relevance of dysregulated genes that are nonorthologous to HSA21 genes; therefore, we cannot account for their specific contribution to observed phenotypes over the Ts1Cje lifespan.

Despite the large experimental evidence of global gene expression dysregulation in postmortem brains from fetuses with DS (Mao et al., 2005; Olmos-Serrano et al., 2016a), our study is the first to describe abnormal global gene expression in the embryonic forebrain of the three most widely used mouse models of DS [i.e. Ts1Cje, Ts65Dn and Dp(16)1/Yey]. The next most comprehensive developmental gene expression studies have focused on cerebellar development in Ts1Cje mice from P0 until P30 (Dauphinot et al., 2005; Laffaire et al., 2009; Potier et al., 2006). Similar to our work, these studies found a consistent upregulation in the trisomic region of MMU16 in Ts1Cje cerebellum. Many other genes were also shown to be affected, reinforcing the fact that global gene dysregulation is occurring in these animals throughout postnatal development. Differentially regulated genes could not be directly compared between our current study and this prior work due to differences in ages and because only ANOVA was used to identify misexpressed genes. Yet, despite these methodological differences, general trends regarding FC magnitude and global gene expression perturbations were consistent. Interestingly, in Laffaire et al. (2009), cells from the external granule layer were dissected and assessed by qRT-PCR; 80% of upregulated MMU16 genes identified by this analysis were also found in our MDG set from Ts1Cje adult cerebellum. This further reinforces our findings and the methods we used to identify dysregulated genes in the cortex, hippocampus and cerebellum of trisomic mice.

#### Ts65Dn

Similar to fetuses with DS, significant abnormalities in somatic and brain growth, pallial expansion and neurogenesis were observed in Ts65Dn embryonic forebrain. In addition, perinatal deficits in corticogenesis and sex-specific developmental milestone delays were apparent in this model. Additionally, learning and memory deficits in adult Ts65Dn mice, including defects in cognitive flexibility identified by the MWM task, mimicked learning and memory phenotypes seen in individuals with DS. Ts65Dn mice did not show altered motor coordination as assessed by the rotarod test, but did show open field and motor-based developmental milestone defects.

The unique gene expression changes in Ts65Dn forebrain might be partly responsible for the developmental phenotypes observed in these mice. Importantly, our gene expression data from developing Ts65Dn embryos identify dysregulated expression of several triplicated genes that are not orthologous to HSA21 (nine MMU17 genes), indicating that these genes might contribute to the prenatal phenotype observed in these mice. Previous work shows that several of these genes play roles in neurological function. For example, mutations in *Arid1b*, a gene that is highly expressed in the developing cortical plate and is upregulated in Ts65Dn embryonic forebrain, are implicated in intellectual disabilities in humans (Sim et al., 2015). In fact, ARID1B is thought to be involved in a neuron-specific chromatin remodeling complex that is associated with the exit of neural progenitors from the cell cycle and their differentiation into postmitotic neurons (Sim et al., 2015). Therefore, upregulation of *Arid1b* might impede cell cycle progression and/or proliferation, contributing in part to the observed decrease in neurogenesis and postmitotic neurons in the Ts65Dn brain. Similarly, *Serac1* is expressed in the IZ at E15.5 and is upregulated in Ts65Dn embryonic forebrain. *Serac1* encodes a phosphatidylglycerol remodeling protein that plays a role in mitochondrial function and intracellular cholesterol trafficking. How upregulation of this gene might contribute to proper neural development is unknown; however, dysregulation in *Serac1* has been linked to mitochondrial-based encephalopathy in humans (Wedatilake et al., 2015). It is, therefore, possible that its dysregulation could impact typical brain development in Ts65Dn mice. Aside from these dysregulated MMU17 genes, our embryonic and adult gene expression analyses identified many unique DEX genes and MDGs in Ts65Dn brains, indicating that gene dosage rescue experiments are needed to more directly assess the role of each of these dysregulated genes in Ts65Dn-specific brain phenotypes. In theory, these uniquely dysregulated genes, along with their downstream effects, might be the underlying molecular precipitants of some of the phenotypes only observed in Ts65Dn brains.

Several prior studies have investigated gene expression in Ts65Dn brains (Kahlem et al., 2004; Saran et al., 2003; Sultan et al., 2007). Importantly, the methodology in those studies differs substantially from methods used in our current work preventing in-depth comparisons of findings. For example, in Saran et al. (2003), RNA from the cerebellum of six euploid and six Ts65Dn mice were pooled prior to analysis, only 7000 probes were used, and average FC values were not gated using stringent statistical methods. Despite these differences, our two studies support the general principle that global gene expression abnormalities exist as a result of the triplicated MMU16 genes. In work by Sultan et al. (2007), eight animals of each genotype were used for qRT-PCR analyses of genes in the cortex, cerebellum and midbrain. The authors found large intersubject variability and generated three categories to identify consistently upregulated genes compared with genes that either overlap with euploid expression or show no specific stratification by genotype. The authors found that nine genes were consistently upregulated in the cerebella of all Ts65Dn mice, and 17 genes were consistently upregulated in all Ts65Dn cortices. Comparing these genes with our microarray screen, we found that all nine genes within the cerebellum are reflected in our MDG list, seven of which were identified as DEX. Additionally, we identified 16 of the 17 genes found in the cortex and 15 were classified as DEX in our study. This high level of similarity between the findings, despite the use of distinct statistical methods and gene expression assays, validates the experimental paradigm that we used, as well as the thresholding methods we employed to gate our data. Lastly, work by Kahlem et al. (2004), which focused primarily on the triplicated region of MMU16 in Ts65Dn mice, identified several pathway perturbations in cortex and cerebellum (along with five other specialized tissues), two of which were identified in our study: (1) signal transduction, and (2) cell-cell communication/extracellular matrix. Thus, despite the differences in experimental paradigms used to assess gene expression in adult Ts65Dn brains, a general concordance exists between findings. Our current work, therefore, validates and substantially expands upon these prior reports. In addition, our study assesses forebrain gene expression during embryonic development and provides information about expression of MMU17 triplicated genes, characterizing two novel aspects of global gene dysregulation in Ts65Dn brains.

#### Dp(16)1/Yey

Based on the increased number of triplicated syntenic genes in Dp(16)1/Yey mice, we expected to observe an exaggerated or at least a similar phenotype to Ts65Dn mice during embryonic development. Our previous demonstration of a lack of forebrain morphogenesis defects in Dp(16)1/Yey mice was unexpected (Goodliffe et al., 2016). In the present study, we employed EdU, a newer cell cycle marker which has increased sensitivity compared with BrdU, to assess neurogenesis (Qu et al., 2011), but again found no prenatal neurogenesis defects in Dp(16)1/Yey embryos at E15.5. Importantly, we showed that maternal trisomy and a hybrid background strain had no impact on growth indices in Dp(16)1/Yey embryos, because Dp(16)1/Yey animals bred using the same breeding scheme as Ts65Dn mice showed no measurable abnormalities in embryonic brain growth phenotypes. Additionally, postnatal analysis of achievement of developmental milestones indicated that, unlike Ts1Cje and Ts65Dn mice, Dp(16)1/Yey neonates did not show deficits in early-acquired milestones. Instead, these mice showed defects only in late-acquired (P15-P21) milestones concomitant with abnormalities in cortical excitatory neuron and IN populations at P15. Furthermore, Dp(16)1/Yey adults showed profound impairments on motor and learning/memory tasks.

Together, these data suggest that while we were unable to measure any abnormalities in the embryonic or early neonatal phenotypes, Dp(16)1/Yey mice still manifested behavioral and histological phenotypes that began around P15 and showed cognitive and motor impairments as adults. This key finding raises important questions about how pre- and postnatal phenotypes relate to one another in mouse models of DS. This also raises corresponding questions about the assumed connections between pre- and postnatal brain phenotypes in humans with DS.

In theory, the Dp(16)1/Yey model should exhibit the most DS-relevant phenotypes owing to the increase in the number of triplicated HSA21 orthologs and the lack of dosage imbalance of non-HSA21 orthologs. This theory is, however, strongly contradicted by the normal neuroanatomical and developmental milestone data, and the low number of DEX genes in these mice. Gene expression analysis shows that the number of DEX genes in Dp(16)1/Yey embryonic forebrain is approximately half of that found in T65Dn and Ts1Cje forebrains. The lack of discernible gene-phenotype relationships in Dp(16)1/Yey points to possible additional contributions from epigenetic and other regulatory elements, including miRNAs and long noncoding RNAs, or perhaps a compensatory mechanism arising from the increased number of triplicated genes.

Unique to the Dp(16)1/Yey embryonic forebrain was the presence of eight miRNAs within the MDG list (i.e. with a FC>1.2). The presence of these marginally dysregulated miRNAs in Dp(16)1/Yey embryonic forebrain might provide an explanation for the lack of atypical prenatal phenotypes in this model and, as such, warrants further study. Even though the number of genes that met the statistical criteria to be designed as DEX genes is low in Dp(16)1/Yey embryonic forebrain, the number of MDGs is higher compared with Ts65Dn and Ts1Cje. Taken together, the differential expression of miRNA and the large number of MDGs with small FCs suggest that slow cumulative gene expression and pathway changes play a role in delaying the onset of cellular and cognitive phenotypes.

Similarly, Dp(16)1/Yey adult brains showed a consistently high number of MDGs in all regions examined. This correlates with an abnormal behavioral phenotype in fear conditioning, MWM and rotarod tests, suggesting that the cumulative effects of subtle gene expression changes can result in severe behavioral deficits, even if the statistical criteria to classify these genes as DEX genes are not fulfilled. These key findings warrant further studies to better understand the molecular mechanisms responsible for the observed abnormalities in the neonatal and adult histological and behavioral phenotypes that seemingly occur without abnormalities in embryonic brain development.

### Comparisons across models

#### Embryonic somatic growth, brain morphogenesis and gene expression

Only Ts65Dn mice had reduced body length and brain size at E15.5. Breeding onto a B6C3Sn hybrid background in the Ts1Cje (euploid dams×trisomic males) and Dp(16)1/Yey (trisomic dams×euploid males) colonies did not induce similar phenotypes in trisomic embryos. Neocortical expansion was also reduced in Ts65Dn mice at E15.5, but was largely unaffected in Dp(16)1/Yey mice. On the other hand, Ts1Cje mice showed a selective increase in the thickness of the IZ of the dorsal germinal zone. Neurogenesis was decreased in the VZ/SVZ of the dorsal pallium of Ts65Dn mice but was unchanged in Ts1Cje and Dp(16)1/Yey mice. Ts65Dn mice also exhibited a consistent increase in mitotic events and progenitor cell numbers within the MGE of the ventral germinal zone. These changes were not observed in Ts1Cje or Dp(16)1/Yey brains. Ts65Dn and Ts1Cje embryonic forebrains had a similar number of DEX genes, with ∼50% mapping to the MMU16 triplicated region in each strain. Several DEX genes in these two models, however, were products of non-HSA21 orthologs that are uniquely aneuploid in each respective strain. Despite having the largest MMU16 triplication, Dp(16)1/Yey embryonic forebrains had the lowest number of DEX genes. Yet, Dp(16)1/Yey brains also had the highest number of MDGs.

In Ts1Cje forebrains, *Dnah11*, a gene found on MMU12, was consistently and markedly upregulated in all regions. We previously determined that the overexpression of *Dnah11* is an artifact of the translocation breakpoint in Ts1Cje mice (Guedj et al., 2015b). Truncation of the gene likely leads to its dysregulation. Importantly, despite its differential expression, *Dnah11* did not play a role in any functional pathways in Ts1Cje embryonic forebrains. However, this does not preclude the fact that it could negatively affect other organs and thus indirectly affect the brain.

Ts1Cje, Ts65Dn and Dp(16)1/Yey embryonic forebrains shared very few dysregulated signaling pathways and cellular processes relating to neurogenesis and brain development. All three strains, however, showed an upregulation in interferon signaling and immune response. Several additional pathways indicated by GSEA and/or DAVID shed light on cellular dysregulation in Ts1Cje, Ts65Dn and Dp(16)1/Yey forebrains that might relate to abnormalities in brain development. As examples, downregulation in neural crest cell development in Ts65Dn mice and downregulation in transcriptional regulation and oxygen handling in Dp(16)1/Yey mice could be functional consequences of dysregulation occurring at the cellular level. Additionally, olfactory receptor activity involving G-protein-coupled receptors was significantly upregulated in both the Ts65Dn and Dp(16)1/Yey embryonic forebrains. Very few studies have investigated the importance of olfactory receptor expression in neurons other than olfactory sensory neurons (Cecchini et al., 2016). However, there is evidence indicating the importance of olfactory recognition of familiar pheromones (i.e. maternal) for feeding in mouse pups and in human infants (Lévy et al., 2004; Schaal, 2010). Also, recent work shows that olfactory function and explicit olfactory memory are severely affected in individuals with DS (Cecchini et al., 2016; Johns et al., 2012). Therefore, as a follow-up study, we are investigating olfactory recognition and contextual olfactory memory in Ts1Cje, Ts65Dn and Dp(16)1/Yey neonates to determine whether these gene expression abnormalities manifest as a DS-related behavioral phenotype.

#### Neonatal cellular populations and developmental milestones

Ts65Dn and Ts1Cje pups exhibited deficits in achieving both early and late developmental milestones. Conversely, Dp(16)1/Yey mice only exhibited deficits in achieving late developmental milestones. Sex differences in achieving milestones were only observed in the Ts65Dn mice, with males showing more delays in a greater number of tasks. Ts65Dn mice exhibited an increase in specific cortical and hippocampal IN subtypes as well as a decrease in cortical excitatory neurons at P15. In contrast, specific subtypes of cortical INs as well as excitatory neurons were decreased in the Dp(16)1/Yey somatosensory cortex, but hippocampal IN populations were unchanged. In Ts1Cje mice, overall cell numbers, inhibitory IN populations and excitatory neurons were unchanged; however, a shift in laminar distribution in neocortical excitatory neuronal populations was observed. These data suggest that alterations in IN specification might be related to the milestone delays in the Ts65Dn and Dp(16)1/Yey models, but that inhibitory neuron defects probably do not underlie the perinatal behavioral deficits in the Ts1Cje mice. Although the most significant changes were again found in the Ts65Dn brains, slight deficits in excitatory neuron number and in laminar specification might underlie the Ts1Cje milestone findings.

#### Adult behavior and global gene expression

Reflexive behavior as assessed by SHIRPA was largely unaffected in all three models. In hippocampal-based tasks, all three models showed CFC abnormalities, but only Ts65Dn and Dp(16)1/Yey mice showed MWM abnormalities. In motor-based tasks, Ts1Cje mice showed both rotarod and open field deficits, Ts65Dn mice showed only open field deficits, and Dp(16)1/Yey mice showed severe rotarod deficits.

Ts65Dn mice exhibited the lowest number of DEX genes in the hippocampus, but the highest number of DEX genes in the cortex, compared with Ts1Cje and Dp(16)1/Yey models. On the other hand, in cerebellar tissue, Dp(16)1/Yey mice showed the lowest number of DEX genes compared with Ts1Cje and Ts65Dn mice. As in embryonic Ts1Cje forebrains, *Dnah11* is consistently and markedly upregulated in all examined regions in adult Ts1Cje brain. In fact, in adult animals, *Dnah11* representation in several functional pathways reinforces its possible impact on gene networks and on other phenotypes. Additional studies on whether dysregulation in *Dnah11* expression impacts brain function are needed, but its role in DS is unlikely.

Despite the fact that Ts65Dn hippocampus had the lowest number of DEX genes, Ts65Dn mice showed pronounced deficits in hippocampal-based tasks. Interestingly, Ts65Dn mice had the highest number of MDGs in the hippocampus and the highest number of upregulated genes overall, suggesting that there could be a link between genes exhibiting small FCs and behavioral deficits observed in hippocampal-based tasks in this model. Additionally, Dp(16)1/Yey whole brains (cortex+hippocampus+cerebellum) exhibited the highest number of MDGs and these mice showed both hippocampal- and motor-based behavioral deficits.

Similar to our findings in the embryonic forebrain, Ts1Cje, Ts65Dn and Dp(16)1/Yey adult brains shared very few dysregulated pathways and cellular processes, but all three showed upregulation in interferon signaling and immune response pathways. This finding, which is confirmed by both GSEA and DAVID analyses, correlates well with recent work assessing proteomic changes in blood samples from people with DS (Sullivan et al., 2017) and another study analyzing interferon-related gene networks in postnatal Ts1Cje brains (Ling et al., 2014). The implications of this correlation are twofold: (1) our gene expression data and downstream analyses reflect significant physiological changes occurring in all three mouse models and confirmed in people with DS; and (2) the link between chronic immune dysregulation and brain function is tenuous in mouse models of DS, because all models exhibited significantly dysregulated gene expression related to the immune response but not all had concomitant brain-specific phenotypes.

### Impact of model-specific cytogenetics on observed phenotypes

Unique to the Ts65Dn mouse model is the existence of a freely segregating marker chromosome, making this model the only aneuploid model of DS assessed here. The assertion that gene dosage, i.e. allelic number of HSA21, underpins DS phenotypes has long governed mechanistic studies aimed at elucidating relationships between particular genes and observed phenotypes (Chakrabarti et al., 2010; Pritchard and Kola, 1999; Rachidi and Lopes, 2008; Roper and Reeves, 2006). This hypothesis that cumulative genetic or epigenetic changes manifest as structural and behavioral deficits might explain the presence of abnormalities in Ts65Dn but not Ts1Cje brains, because there are more triplicated genes in Ts65Dn. However, this is largely contradicted by the lack of an abnormal brain phenotype in Dp(16)1/Yey embryos and neonates, which have 13% more triplicated HSA21 orthologs than Ts65Dn mice. As somatic and brain growth abnormalities are measurable during gestation in human fetuses with DS, we would expect that DS phenotypes recapitulated by Ts65Dn mice would be exacerbated as the number of triplicated genes within the MMU16 syntenic region increases [i.e. in Dp(16)1/Yey mice]. Since our data show that this is not the case, the gene dosage hypothesis alone does not explain the sequelae of HSA21 triplication.

Additional factors that might contribute to the atypical phenotypes seen in DS are the physical state of triplicated chromatin and the presence of an additional chromosome, or aneuploidy, in 95% of cases of DS (Shin et al., 2010). The amplified developmental instability hypothesis states that most DS phenotypes are a result of a nongene-specific disturbance in chromosomal balance, leading to disrupted homeostasis (Pritchard and Kola, 1999). This hypothesis suggests that there is a common mechanism underlying abnormal phenotypes observed in people with different aneuploidies (i.e. trisomies 21, 18 and 13), while simultaneously accounting for interindividual variation among people with DS (Pritchard and Kola, 1999). Notably, several studies have shown that trisomic mice, regardless of which chromosome is triplicated, exhibit stunted embryogenesis and widespread hypoplasia compared with euploid littermates. This includes mice with individual triplications in all autosomes (MMU1*-Mmu*19) as well as the Ts16 mouse model of DS, in which the full MMU16 chromosome is triplicated (Gearhart et al., 1986; Haydar et al., 1996, 2000; Williams et al., 2008). More in-depth cellular studies in Ts65Dn mice have also shown that Ts65Dn embryos and neonates exhibit decreased proliferation and elongation of the cell cycle in the brain and in peripheral tissue (Chakrabarti et al., 2007; Contestabile et al., 2009a,b, 2007). Furthermore, gene expression analysis in these mice pinpointed a specific decrease in regulators of G(2)/M and G(1)/S cell cycle transition (Contestabile et al., 2007). Thus, we suggest that cell cycle aberrations caused by the presence of an additional chromosome in Ts65Dn mice might be necessary, in conjunction with the gene dosage imbalance, for the induction of abnormalities in embryonic brain morphogenesis. This is a possible explanation for why Ts1Cje and Dp(16)1/Yey embryos show no apparent DS-related prenatal brain development deficits in our study. Thus, the combination of abnormal gene dosage and developmental instability, resulting from the aneuploidy itself, could be a major modulator of abnormal brain phenotypes in the mouse. This new combinational ‘gene dosage/developmental instability’ theory can be further substantiated by limited studies showing that people with a translocation of the long arm of chromosome 21, who have triplication of HSA21 genes but no aneuploidy, exhibit less severe phenotypes compared with the 95% of people with DS who have aneuploidy (Chandra et al., 2010; Prasher, 1995). Additional large-scale studies are necessary to fully characterize molecular, histological and cognitive differences in these two affected human populations.

### Comparison of models to human phenotypes

Spatiotemporal physiological changes are well documented in people with DS and can be used as a metric to identify suitable models for basic and translational studies (Bahado-Singh et al., 1992; Cardoso et al., 2015; Guidi et al., 2008; Haim et al., 2009; Larsen et al., 2008; Lott, 2012; Nilholm, 1999; Vicari et al., 2013; Winter et al., 1998; Wisniewski and Kida, 1994; Wisniewski et al., 1984). Our work shows that Ts65Dn mice model the symptomatic arc identified in people with DS well, but not perfectly: neurogenesis defects were observable pre- and postnatally, delays in developmental milestones were present at birth, and learning and memory deficits were seen throughout adulthood. In addition, we show that Ts65Dn males exhibit more profound deficits in developmental milestones compared with females, reproducing some aspects of the sex differences observed in males and females with DS (Kittler et al., 2004; Määttä et al., 2006; Marchal et al., 2016). Furthermore, age-dependent decline in performance has previously been reported in these mice (Belichenko et al., 2007; Costa et al., 2010; Holtzman et al., 1996; Olmos-Serrano et al., 2016b; Reeves et al., 1995; Xing et al., 2016). Importantly, despite the prenatal neurogenesis deficits, we did not consistently observe microcephaly, a hallmark of DS, in the Ts65Dn brain.

On the other hand, until now, Ts1Cje and Dp(16)1/Yey mice had not undergone the rigorous tests to which Ts65Dn mice were subjected. Previous data related to these two models were scarce and somewhat contradictory. Here, we showed that Ts1Cje mice exhibit mild abnormalities in embryonic and perinatal forebrain histogenesis that do not recapitulate what has been reported in Ts65Dn or in brains of individuals with DS. In addition, while Ts1Cje neonates show deficits in both early- and late-acquired developmental milestones, Ts1Cje adults exhibit only mild deficits in some of the behavioral tasks. The discrepancies between the histological findings and the developmental milestone deficits in Ts1Cje pups are unresolved. However, the differences between the developmental milestone data and the mild adult behavioral deficits found in Ts1Cje mice might be related to the mortality of the most highly-affected animals in the first several weeks of life (Ferrés et al., 2016). Similarly, forebrain histogenesis in embryonic Dp(16)1/Yey mice appears normal, yet inhibitory and excitatory neuron population deficits are present in P15 Dp(16)1/Yey mice. Despite normal performance on early-acquired developmental milestones, Dp(16)1/Yey neonates show deficits in late-acquired developmental milestones and in motor and learning and memory tasks as adults. This delayed onset of behavioral phenotypes might be caused, in part, by the small, but cumulative, changes in gene expression or the postnatal changes in neuron number detected in the somatosensory cortex. More work is necessary to better understand how and why these postnatal brain abnormalities arise and to uncover the underlying etiology of observed behavioral deficits in adult Dp(16)1/Yey animals. Taken together, our results suggest that Dp(16)1/Yey mice could be useful for investigating postnatal brain and behavioral abnormalities that are not reliant on aneuploidy and arise independently from prenatal corticogenesis deficits.

### Future work

Until now, no experiments have specifically addressed the contribution of the nonsyntenic genes triplicated in the Ts65Dn mice to the observed phenotypes. Here, we show that some of these genes are uniquely affected in Ts65Dn brains during development and in adulthood. Determining whether the abnormalities in Ts65Dn are caused by the triplication of these nonsyntenic MMU17 genes requires a new model lacking these unrelated genes, perhaps generated by gene editing technologies. We believe that the data strongly indicate that such a model is now necessary. Similarly, seven genes on MMU12 are monosomic in Ts1Cje, leading to dysregulated expression of those genes; we cannot fully determine their contribution to Ts1Cje-specific phenotypes but have measured decreases in their expression by microarrays. Additionally, Ts65Dn is the only model of aneuploidy assessed in the current study. Determining the specific contribution of the additional chromosome to observed phenotypes is pivotal to a better understanding of DS. This could be accomplished by comparing Ts65Dn to its genocopy Ts2Cje (Villar et al., 2005), which contains the same triplicated genes but does not have an extra, freely segregating chromosome. The comprehensive set of methods and analyses used in this study could provide a roadmap for these future investigations. Lastly, the effect of the duplication and chromosomal elongation in Dp(16)1/Yey on chromatin state is unknown, but consequent changes in epigenetic regulation of gene expression could play a role. Alternatively, it is possible that some of the additionally triplicated genes in this model compensate for or diminish the abnormalities seen in other models and/or that a combination model of all HSA21 orthologs is necessary for the emergence of DS-related phenotypes in mice that contain the extra gene copies as an elongation of an existing chromosome not as an aneuploidy (Zhang et al., 2014).

This study provides a baseline for additional comparative studies, especially as new mouse models of DS are developed. Gene expression data from human brains point to key biological processes that are also disturbed in people with DS such as myelination, synaptogenesis and neuroinflammation (Olmos-Serrano et al., 2016a). These processes can be further explored and then targeted individually or in combination for treatment. Although we focused only on forebrain development, abnormalities in subcortical, cerebellar and brainstem regions have been reported in people with DS and in some mouse models. These brain structures undoubtedly play a role in behavioral phenotypes associated with DS. Lastly, we focused on molecular, structural and behavioral abnormalities in Ts1Cje, Ts65Dn and Dp(16)1/Yey mice. Much work is still needed to assess subcellular, cellular and electrophysiological function in these mice.

### Conclusions

Our data show widespread and unexpected fundamental differences in gene expression, corticogenesis and behavior in the three most common mouse models of DS. Furthermore, our data raise important questions about the downstream anatomical or functional consequences of different numbers of dysregulated genes. Our results also challenge previously held assumptions regarding correlations between embryonic brain development and later behavioral or cognitive abnormalities.

Ts65Dn mice recapitulate most of the neuroanatomical and behavioral alterations typically found in people with DS at different ages. The triplication of ∼60 nonrelevant genes in this model might, however, influence some of these observed changes. Notably, because the relationship between prenatal and postnatal phenotypes in DS is still not well understood, the Ts1Cje and Dp(16)1/Yey models could be useful for elucidating cognitive or behavioral changes that occur in the absence of significant prenatal effects on brain development and in testing spatiotemporally restricted therapeutic interventions.

On the other hand, our work clearly highlights the fact that, based on genetic construction, gene expression, histology and behavior, the Ts1Cje, Ts65Dn and Dp(16)1/Yey strains all have limitations in accurately modeling the human condition. Thus, there is a crucial need for the generation of additional models that better recapitulate DS phenotypes and genetics.

## MATERIALS AND METHODS

We developed a comprehensive standardized protocol to evaluate the molecular, cellular and behavioral phenotypes in the Ts1Cje, Ts65Dn and Dp(16)1/Yey mouse models of DS at embryonic, neonatal and adult stages. This enabled a direct comparison of models, as well as comparison with the known phenotypic changes in individuals with DS. While some of the subsets of data on individual models have been previously published by our groups (Chakrabarti et al., 2010, 2007; Goodliffe et al., 2016; Guedj et al., 2015a,b; Olmos-Serrano et al., 2016b), new tests were added, new gestational ages were studied, and all prior data were newly re-analyzed to provide a consistent comparison. For completeness, all methods are described here.

### Animal breeding and genotyping

#### Animal breeding

All murine experiments were conducted according to international ethical standards and approved by the Institutional Animal Care and Use Committees (IACUC) of Boston and Tufts Universities. Animals were housed in cages with standard bedding and a nestlet square. Rodent chow and water were available *ad libitum*. The colonies were maintained on a 12:12 light/dark cycle, with lights on at 07:00.

Studies were performed at three different life stages: (1) embryonic: E15.5 for global brain gene expression, gross anatomy and neuroanatomy/neurogenesis experiments; (2) neonatal: between birth and P21 for neonatal behavior and excitatory/inhibitory neuronal density experiments; and (3) adult: between 3 and 7 months for behavioral and cerebellar, hippocampal and cortical gene expression experiments.

B6.Cg-T(12;16)1Cje/CjeDnJ mice (Ts1Cje; stock number 004838), B6EiC3Sn.BLiA-Ts(17^16^)65Dn/DnJ (Ts65Dn; stock number 005252) mice and B6129S-Dp(16Lipi-Zfp295)1Yey/J (Dp(16)1/Yey; stock number 013530) mice were purchased from Jackson Laboratory (Bar Harbor, ME). Ts65Dn female mice were bred with B6EiC3Sn.BLiAF1/J (F1 hybrid; stock number 003647) males. Ts1Cje and Dp(16)1/Yey males were bred with C57BL/6J (Jackson Laboratory) or C57BL/6N females (Charles River Laboratories, Wilmington, MA). To test the contribution of background strain to the observed phenotypes, both Dp(16)1/Yey and Ts1Cje females were bred with C3Sn.BLiA-*Pde6b^+^*/DnJ males and the resultant progeny were bred the following ways: (1) Dp(16)1/Yey B6129SC3Sn.BLiAF1/J F1 hybrid females were bred with B6EiC3Sn.BLiAF1/J F1 hybrid males to generate B6C3Sn-Dp(16Lipi-Zfp295)1Yey/NJ in a manner that mimics Ts65Dn breeding; and (2) Ts1Cje B6C3Sn.BLiAF1/J F1 hybrid males were bred with euploid B6EiC3Sn.BLiAF1/J F1 hybrid females to generate B6C3Sn.Cg-T(12;16)1Cje/CjeDnJ mice.

In all experiments, trisomic mice were compared with their euploid littermates. This was done precisely to avoid comparing trisomic mice of one strain with euploid mice from another strain. Specifically, it is well known that baseline differences exist between the various substrains of C57BL/6 mice, as well as those mice on a C57BL/6XC3Sn hybrid background (Bryant et al., 2008; Simon et al., 2013). Therefore, to ensure that any phenotypic differences arose only from trisomy, and not from genetic differences in background strains, we first compared trisomic animals with euploids of that strain, then evaluated the presence, absence or magnitude of phenotypic differences across strains, to compare and contrast the three mouse models. Importantly, in typical phenotyping studies, genetically manipulated mice are compared only with unaffected mice of their same background strain. This is the exact same paradigm that we utilized in our cross-model comparison.

Additionally, although in some studies we reported results as a percentage of that of euploid, statistical analyses of these studies ensured that any baseline variability in the control group was accounted for when reporting a given phenotypic abnormality.

#### Genotyping

Phenol/chloroform DNA extraction was performed on embryonic limb buds or postnatal tail clippings after digestion with proteinase K (Denville Scientific, Holliston, MA). Genotyping and sex determination were performed by PCR using primers specific for the Ts1Cje (Olson et al., 2004), Ts65Dn (Reinholdt et al., 2011) or Dp(16)1/Yey (Goodliffe et al., 2016) translocation breakpoints and the *SRY* region along with an internal positive control (Table S27).

### Tissue collection

#### Embryonic brain collection: gene expression

Breeding pairs were established so that vaginal plugs could be checked twice daily. The presence of a vaginal plug was designated as E0.5. A 10% weight gain at E10 was used to confirm pregnancy (Johnson et al., 2010). Pregnant dams were euthanized at E15.5. Embryos were extracted, identified as E15.5 using Theiler staging, and decapitated in ice-cold 1× phosphate-buffered saline (PBS) containing RNAprotect^®^ cell reagent (Qiagen, Germantown, MD). Embryonic forebrains were rapidly removed and brain hemispheres dissected on a cold platform and snap frozen in liquid nitrogen before storage at –80°C.

Five to six mice per group were used for microarray analyses (Table S28), and eight mice were used for qRT-PCR analysis (five of which were original samples used in microarrays, and three were new samples).

#### Embryonic brain collection: histology/neuroanatomy

E15.5 embryos were collected and fixed for 24 h in 4% paraformaldehyde (PFA) at 4°C. Embryos were then washed three times in 1× PBS and brains were dissected for gross measurements. After measurements were completed, fixed brains were placed in 30% sucrose for 16-36 h at 4°C then frozen in Optimal Cutting Temperature Compound (OCT; Sakura, Torrance, CA). Tissue blocks were stored at −80°C until use. Serial coronal sections (16 μm) were cut using a cryostat, mounted on Superfrost^®^ Plus charged slides (ThermoFisher Scientific, Waltham, MA), and stored at −80°C.

#### Neonatal brain collection: histology

P15 male mice were anesthetized with a xylazine/ketamine cocktail according to IACUC regulations. Mice were transcardially perfused with 4% PFA, and brains were extracted and post-fixed for 16 h in 4% PFA at 4°C. Brains were then prepared as described above.

The number of mice per group were as follows: (1) Ts1Cje – six euploid, six trisomic; (2) T65Dn – four euploid, four trisomic; (3) Dp(16)1/Yey – five euploid, four trisomic (Table S28).

#### Adult tissue collection: global gene expression

For adult gene expression studies, 6- to 7-month-old male mice were anesthetized with 2.5% isoflurane in a 3/7 O_2_/N_2_O mixture and euthanized by decapitation. Brains were removed from the skull and dissected on a cold platform. Cerebral cortex, hippocampus and cerebellum were dissected and snap frozen in liquid nitrogen and stored at −80°C.

Five mice per group were used for microarray analyses (Table S28), and six to seven mice were used for qRT-PCR analysis (five of which were original samples used in microarrays, and the rest were new samples).

### Gene expression studies using microarrays

For gene expression studies, total RNA was isolated from the developing forebrain using the RNA II kits following the manufacturer's instructions (Macherey-Nagel, Bethlehem, PA). RNA was processed and hybridized on a GeneChip^®^ Mouse Gene 1.0 ST array as described previously (Guedj et al., 2016). Statistical analyses were carried out on the normalized data using R software (version 3.1.2 or later). Normalization was performed using the robust multichip average algorithm and the MBNI custom CDF version #15 for the mouse gene 1.0 ST array. Normalization output consisted of data for 21,225 probe sets, each corresponding to unique Entrez Gene IDs. Gene expression data from Ts1Cje, Ts65Dn and Dp(16)1/Yey tissue were compared with those from their respective euploid littermates using an unpaired Student's *t*-test. *P*-values for the combined comparisons included in this study were jointly corrected for multiple testing using the Benjamini-Hochberg FDR (Benjamini and Hochberg, 1995). We used different FDR cut-offs (<5, <10 and <20%) to identify DEX genes in trisomic mice compared with their euploid littermates. For functional pathway analysis, we used DAVID (Huang et al., 2009). Gene Ontology (GO) or Kyoto Encyclopedia of Genes and Genomes (KEGG) terms were considered significantly enriched if the DAVID *P*-value was <0.05. To increase the depth of our pathway analysis, we generated a gene list (MDGs) by gating FC (FC cut-off of >1.20 for the upregulated genes and <0.8 for the downregulated genes) in genes that showed a statistically significant dysregulation with a *P*<0.05. In addition, we performed whole-transcriptome pathway analysis using GSEA as described in Guedj et al. (2015b), using gene set collections downloaded from the Molecular Signatures Database (MSigDB; www.broadinstitute.org/gsea/msigdb/) v5.1. Gene sets (for pathways or other functional terms) with *P*<0.05 were considered significantly dysregulated.

### qRT-PCR validation of microarray data

For qRT-PCR validation, RNA prepared from embryonic E15.5 forebrains, adult cerebral cortex and adult hippocampus was converted to complementary DNA (cDNA) using an Ambion RETROScript kit according to the manufacturer's instructions (Thermo Fisher Scientific, Waltham, MA). First, 100 ng of cDNA was used for the multiplex qRT-PCR reaction, combining a target gene and one of the two housekeeping genes *Gapdh* and *Hprt*. The target genes chosen for validation were *Hspa13* [two copies in Ts1Cje and Ts65Dn mice and three copies in Dp(16)1/Yey mice], *App* [two copies in Ts1Cje mice and three copies in Ts65Dn and Dp(16)1/Yey mice], *Ttc3* (three copies in all three models), and *Rfx5* (two copies in all three models) (Table S29). Multiplex qRT-PCR was performed using TaqMan Multiplex Master Mix in MicroAmp™ EnduraPlate™ Optical 384-Well Clear Reaction Plates according to the manufacturer's instructions (Thermo Fisher Scientific). Amplification was conducted using a QuantStudio 7 Flex Real-Time PCR System (Thermo Fisher Scientific). Data analysis was performed using the Expression Suite Software to determine the relative quantity of each transcript of interest (Thermo Fisher Scientific).

### Neuroanatomical studies

#### Embryonic brains

##### Gross measurements

All measurements of brain growth were conducted as previously described (Chakrabarti et al., 2007; Goodliffe et al., 2016). Briefly, embryos were imaged using an Olympus MVX10 brightfield microscope coupled with a Zeiss AxioCam MRc camera. Somatic and gross brain measurements were determined using Axiovision software (Zeiss). All embryo crown-rump lengths were measured from the top of the head to the base of the tail. For gross brain measurements, brains were removed and cleared of all other tissue and the maximal rostrocaudal and mediolateral lengths of each telencephalon were measured.

The number of mice per group were as follows: (1) Ts1Cje – 11 euploid, 13 trisomic mice; (2) T65Dn – 20 euploid, seven trisomic mice; (3) Dp(16)1/Yey – 19 euploid, 26 trisomic mice; (4) Ts1Cje B6C3Sn hybrid background – 18 euploid, six trisomic mice; (5) Dp(16)1/Yey B6C3Sn hybrid background – nine euploid, seven trisomic mice.

##### Pallial expansion measurements

Embryonic brain sections were either stained with 1 mM TO-PRO^®^-3 (Thermo Fisher Scientific) according to the manufacturer's protocol or with DAPI (Vector Laboratories, Burlingame, CA). After staining, slides were mounted with Vectashield (Vector Laboratories) and sealed. Slides were then scanned with an LSM710 Zeiss confocal microscope as described below. Using anatomical markers, analysis was always constrained to the future somatosensory cortex for consistent comparison between animals. The entire thickness from the ventricle to the pia within the dorsal pallium was measured. Additionally, the pallium was subdivided into distinct germinal layers – the VZ/SVZ, the IZ and the SP/CP – based on the shape and density of nuclei. The thickness of each subdivision was then also measured.

The number of mice per group were as follows: (1) Ts1Cje – six euploid, six trisomic mice; (2) T65Dn – nine euploid, nine trisomic mice; (3) Dp(16)1/Yey – 10 euploid, 11 trisomic mice.

##### Neurogenesis assays

Pregnant dams were injected with a 50 mg/kg body weight solution containing a thymidine nucleoside analog known as EdU (Thermo Fisher Scientific). These females were euthanized 24 h postinjection at E15.5 and embryonic tissue collection proceeded as described above. A modified protocol was established to stain for EdU utilizing Click-iT^®^ technology (Thermo Fisher Scientific, described in detail in Goodliffe et al., 2016).

The number of mice per group were as follows: (1) Ts1Cje – six euploid, six trisomic mice; (2) T65Dn – nine euploid, nine trisomic mice; (3) Dp(16)1/Yey – 10 euploid, 11 trisomic mice.

##### Immunohistochemistry

When necessary, depending on the tissue penetrance and antigen recognition ability of the antibody used, antigen retrieval was performed by microwaving slides in 10 mM sodium citrate buffer for 1 min at maximum power, followed by 10 min at minimum power. Slides were then washed in 1× PBS and incubated in blocking solution (5% normal donkey or normal goat serum, 0.2% Triton^®^ X-100 in PBS) for 1 h at room temperature. This was followed by incubation in primary antibody overnight at room temperature. Slides were washed in 1× PBS and incubated with secondary antibody solution for 1 h at room temperature. Slides were mounted in Vectashield with DAPI (Vector Laboratories). Primary antibodies used were as follows: rabbit anti-oligodendrocyte transcription factor 2 (1:300, AB9610, Millipore, Burlington, MA), rabbit anti-phosphorylated histone 3 (1:500, 06-570, Millipore), rat anti-somatostatin (1:50, MAB354, Millipore), rabbit anti-parvalbumin (1:1000, PV25, Swant, Marly, Switzerland), rabbit anti-calretinin (1:1000, Swant, 769913), and rabbit anti-Tbr1 (1:1000, gift from the Hevner laboratory, University of Washington School of Medicine, Seattle, WA). The following secondary antibodies were used: (1:250 dilution, Thermo Fisher Scientific): donkey anti-rabbit 555 (A31572), goat anti-rabbit 546 (A11035), goat anti-rabbit 488 (A11008) and goat anti-rat 488 (A11006).

#### Confocal microscopy

Using a combination of DAPI and TOPRO-3 staining, we demarcated the different germinal layers within the dorsal telencephalon at the level of the future somatosensory cortex. We used staining pattern as well as nuclear shape to subdivide the developing pallium into three zones: (1) the VZ/SVZ, (2) the IZ and (3) the SP/CP. All sections were imaged using a Zeiss LSM 710 confocal microscope system. Sixteen 1-μm thick z-stacks of each region of interest were acquired using a 20× objective (NA 0.80).

#### Cell population analysis in embryonic and postnatal tissue

Labeled cells were either automatically counted using Volocity software (Perkin Elmer, Waltham, MA) following manual validation of randomly selected samples, or manually counted using ImageJ (https://imagej.nih.gov/ij/) and LSM Image Browser software. As a general rule, all cells within an entire area of interest were counted so that unbiased stereological techniques were unnecessary. Where noted, cell distribution was determined by measuring cell positions with reference to the ventricular wall. Analyses were limited to the dorsal pallium and medial ganglionic eminence at the level of the future somatosensory cortex in embryonic samples, and to the level of the somatosensory cortex and dorsal hippocampus in postnatal animals. DAPI staining was used to determine neocortical and hippocampal layer boundaries. Both females and males were included in prenatal analyses but postnatal analyses were limited to males only to allow for comparison of the current work with previously published literature.

### Behavioral studies

All behavioral experiments were conducted in the light phase, between 08:00 and 13:00. To minimize olfactory cues from previous trials, each apparatus was thoroughly cleaned with Sani-Cloth Plus (PDI Healthcare, Hamilton, NJ). For each day of testing, mice were left in their home cages in the room used for the experiment at least 1 h prior to the onset of the study. Pups were placed with nesting material in a bowl positioned on a heating pad at 37°C. The MWM task was the last experiment in the series. For all experiments, the investigator was blind to the genotype.

#### Neonatal developmental milestones

Male and female Ts1Cje, Ts65Dn, Dp(16)1/Yey mice and their euploid littermates were tested as previously described (Fox, 1965; Hill et al., 2008; Olmos-Serrano et al., 2016b). Briefly, a set of neonatal behavioral tests was chosen to measure different sensory and motor development parameters in neonatal mice from birth until P21. These tests measured four broad categories of perinatally acquired skills: (1) body righting and coordination (surface righting, air righting and negative geotaxis), (2) motor strength (cliff aversion and forelimb grasp), (3) sensory system maturation (rooting, auditory startle, ear twitch and eye opening) and (4) extinction of rotatory behavior (open field). The amount of time to achieve a developmental milestone (latency) and the presence or absence of a reflex was recorded and analyzed by a single experimenter who was blind to animal genotypes. In total, 255 neonatal mice were tested: 32 Ts1Cje and 64 euploid littermates, 23 Ts65Dn and 34 euploid littermates, and 30 Dp(16)1/Yey and 72 euploid littermates.

#### Adult behavior

The SHIRPA behavioral screen, open field, rotarod, contextual fear conditioning and MWM tests were used to investigate adult behavior. In total, 87 male adult mice were tested: 13 Ts1Cje and 15 euploid littermates, 12 Ts65Dn and 12 euploid littermates, and 17 Dp(16)1/Yey and 18 euploid littermates (Table S28).

##### SHIRPA primary behavioral screen

The SHIRPA screen enables a rapid semi-quantitative assessment of multiple primary body functions, including those that relate to muscle and motor neuron, spinocerebellar, sensory, neuropsychiatric and autonomic systems (Rogers et al., 1997). The experimenter was blind to the genotype, and the performance of each mouse was scored according to the scale provided in Table S11.

##### Exploratory behavior and spontaneous locomotor activity

Exploratory behavior and locomotor activity were assessed using the open field test as described previously (Deacon, 2006). Briefly, the mouse was placed in an open field arena consisting of a white opaque plastic box 40 cm (L)×40 cm (W)×40 cm (H) divided into a center zone measuring 20 cm (L)×20 cm (W)×20 cm (H) and periphery. Exploratory behavior was tracked during a 60-min unique trial using the Ethovision 10.5 animal tracking system (Noldus, Leesburg, VA). The total distance traveled (cm) in the center versus periphery, as well as the average velocity (cm/s), was analyzed for each genotype. Data were collected as time bins of 20 min and as total time over the course of the experiment.

##### Motor coordination

Motor coordination was investigated using the rotarod test (Med Associates, Fairfax, VT) using two different protocols (fixed speed protocol on day 1 and accelerating speed protocol on day 2). Prior to testing with the fixed speed protocol on day 1, each mouse was given two 120 s practice sessions at 16 RPM. After practice, mice were tested at three different fixed speeds (16, 24 then 32 RPM) for two 120 s trials at each speed and with an intertrial interval of 15 min. On day 2, mice were tested in two trials under conditions of increasing difficulty in which the speed of the rotation gradually increased from 4 RPM to 40 RPM over a 5 min period. The latency (in seconds) to fall was recorded and analyzed for each mouse. Euploid littermates of Ts65Dn mice performed poorly compared with other strains of euploid mice. We therefore pooled two cohorts of euploid C57Bl6/C3HSn mice to increase the number of subjects and eliminate confounds.

##### Hippocampal-dependent contextual memory

Hippocampal-dependent memory was analyzed using the fear conditioning test in a conditioning chamber containing a stainless-steel grid floor, an electric aversive stimulator and a house light. This chamber is enclosed within a sound attenuating cubicle with an exhaust fan (Med Associates). On day 1 (training session), each mouse was individually placed for 5 min into the conditioning chamber and allowed to explore freely (habituate) for 180 s. Following exploration/habituation, two mild foot shocks (0.5 mA for 2 s) were administered at 180 s and 240 s. On day 2 (testing session), the mice were placed into an identical conditioning chamber for 5 min with no foot shocks. Each mouse was monitored for freezing (fear) behavior. The extent of (or percentage of time spent) freezing, was analyzed in bins of 60 s and as a total over the course of the experiment using the Freeze View software (Med Associates). These measurements were used as a proxy of the animal's memory of a noxious stimulus.

##### Hippocampal-dependent spatial memory

Hippocampal-dependent spatial memory was analyzed using the MWM test in a 125-cm diameter circular as described previously (Olmos-Serrano et al., 2016b). Mice were trained using an extended protocol containing the following sequence of trials: cued, hidden platform, probe trial, reversal platform and a final probe trial. Each trial lasted for a maximum period of 60 s after which the mouse was guided to the platform and allowed to recover for 15 s before being gently removed by the experimenter. Twenty-four hours after the hidden platform and the reversal platform training sessions, each mouse was subjected to a probe trial to test reference memory. During this test, the platform was removed and mice were allowed to swim once freely for 60 s. Video tracking was performed using Ethovision software (Noldus Actimetrics, Wilmette, IL). Latency to reach the platform, swimming speed, total distance, time spent in the center versus periphery, as well as the time spent in each quadrant were recorded and analyzed. All groups were tested using an identical protocol differing only in the number of days in each phase of testing. Testing ended when (1) trisomic animal performance matched euploid animal performance, or (2) performance in the trisomic experimental group plateaued, indicating a lack of ability to match euploid performance. Importantly, our extended MWM paradigm gave us the ability to qualify whether deficits in spatial learning and memory in trisomic mice are permanent (Stasko and Costa, 2004) or merely delayed (Olmos-Serrano et al., 2016b). We also utilized the elongated testing period to diminish confounding factors that would impact the interpretation of MWM results (Vorhees and Williams, 2006) such as thigmotaxic behavior, jump-offs, and swim-overs previously reported in Ts65Dn mice (Olmos-Serrano et al., 2016b). Additionally, we employed a platform reversal phase to uncover additional cognitive defects (Olmos-Serrano et al., 2016b). Studies in a variety of mutant mouse models with drug treatments that show small or even no difference during hidden platform testing have found significant deficits during reversal training (Vorhees and Williams, 2014).

### Statistical analyses

For all histological and immunohistochemical assessments, unpaired Student's *t*-tests were performed to determine statistical significance between trisomic animals and their euploid controls. All data points were included except those deemed as outliers using Tukey's boxplot method. For behavioral studies, parametric *t*-test or two-way repeated measure ANOVA and post hoc Tukey test were used for normal distributions. Nonparametric Mann–Whitney and Kruskal–Wallis tests were used if values did not follow a normal distribution. For developmental milestone analyses, nonparametric Mann–Whitney and Wilcoxon signed-rank tests were used for single and repeated measures, respectively, to determine significant differences between groups. Fisher’s exact test was used to determine differences between data points. Statistical significance was reached with a *P*-value <0.05.

## Supplementary Material

Supplementary information

First Person interview
