# Supplementary material for: Lifespan analysis of brain development, gene expression and behavioral phenotypes in the Ts1Cje, Ts65Dn and Dp(16)1/Yey mouse models of Down syndrome
Source: Dis Model Mech. 2018 Jun 12;11(6):dmm031013. doi: 10.1242/dmm.031013 (PMC6031353; doi:10.1242/dmm.031013)
Supplement: Supplementary information [file dmm-11-031013-s1.pdf]

First person – Nadine Aziz

First Person is a series of interviews with the first authors of a selection of papers published in Disease Models & Mechanisms, helping early-career researchers promote themselves alongside their papers. Nadine Aziz is first author on 'Lifespan analysis of brain development, gene expression and behavioral phenotypes in Ts1Cje, Ts65Dn and Dp(16)1/Yey mouse models of Down syndrome', published in DMM. Nadine is a Postdoctoral Associate in the lab of Tarik Haydar at Boston University School of Medicine, Boston, USA, investigating the processes that govern human brain and spinal cord development and how changes in these tightly-regulated processes relate to developmental disabilities, in particular Down syndrome.

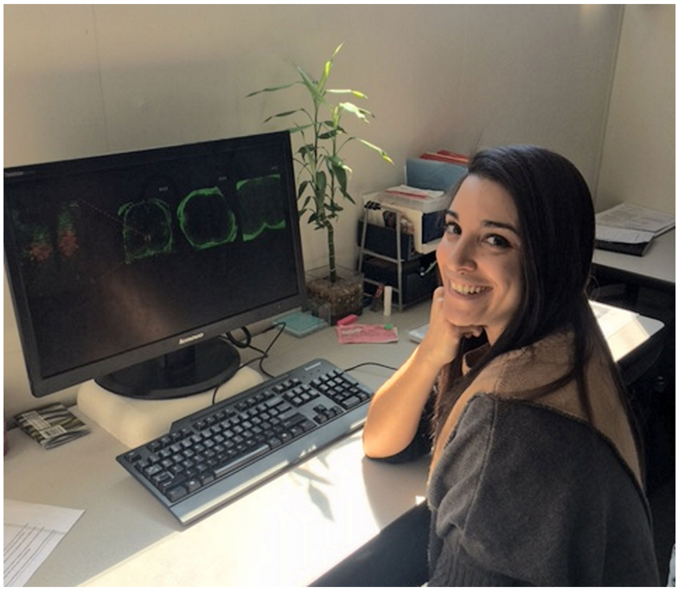

Nadine Aziz

How would you explain the main findings of your paper to non-scientific family and friends?

The main findings of our research indicate that the mouse models that are currently in use to study brain changes in Down syndrome have some profound limitations. In this work, we used multiple approaches to identify and assess brain function on a molecular, cellular, structural and functional level at key periods over the lifespan of three genetically distinct mouse models of Down syndrome. Although these three models have been used almost interchangeably in Down syndrome-related research, our comprehensive screen identified more differences than commonalities between the models. Down syndrome is also known as trisomy 21, which means that there are three copies (triplication) of all or part of chromosome 21 in affected humans, instead of the usual two copies. While each mouse model was genetically engineered to contain a triplicated segment of mouse genes that are analogous to human chromosome 21 genes, we discovered that the number of triplicated genes, coupled with the way in which these genes are expressed, affect the manifestation of Down syndrome-related characteristics. We also identified complex changes in brain anatomy that challenge the notion that prenatal brain developmental changes are directly linked to postnatal brain and cognitive impairments. Lastly, we provided extensive data that can help identify how each mouse model may be best used in Down syndrome research. All in all, our work represents the first side-by-side comparison of widely used mouse models of Down syndrome. The findings challenge the utility of the existing mouse models, identify serious considerations for the development of a new mouse model, and provide a standard screen by which all future models and therapies can be rigorously and reproducibly assessed.

What are the potential implications of these results for your field of research?

The results from our study identify profound limitations in existent mouse models of Down syndrome and indicate the pressing need for the development of a new mouse model. The data also identify multiple novel aspects of cytogenetics and brain development that challenge currently held beliefs in the field. Therefore, the study challenges the current status quo of Down syndrome research and encourages innovation in the engineering of a new model, and rigor and collaboration in effectively assessing any mouse models and their utility in translational studies.

Nadine Aziz's contact details: The Laboratory of Neural Development and Intellectual Disorders, Boston University School of Medicine, Boston, MA 02118, USA.
E-mail: naziz@bu.edu

What are the main advantages and drawbacks of the model system you have used as it relates to the disease you are investigating?

> **"With current advanced gene editing technologies, we can now generate a mouse model that is more genetically similar to people with Down syndrome and extensively assess hallmark phenotypes to validate this mouse model."**

There are many advantages to using mouse models to study complex diseases. Chief among them is the ability they provide investigators to study these diseases and their potential treatments on a much deeper level, which cannot be achieved using human samples alone. The three mouse models that we analyzed, Ts1Cje, Ts65Dn and Dp(16)1/Yey, have contributed tremendously to our knowledge of neurological development and function in Down syndrome. However, due to technological limitations at the time of their engineering, certain drawbacks were unavoidable. With current advanced gene editing technologies, we can now generate a mouse model that is more genetically similar to people with Down syndrome and extensively assess hallmark phenotypes to validate this mouse model. Lastly, mouse model studies should be combined with human-based cellular studies, i.e. patient-specific iPSC-

derived cells or organoids, to more holistically test cellular and molecular changes and their potential therapies, before embarking on costly clinical trials.

Describe what you think is the most significant challenge impacting your research at this time and how will this be addressed over the next 10 years?

The most significant challenge impacting our research is the lack of an open and continuous dialogue between the Down syndrome research community and the patient/caregiver community. Historically, government funding for Down syndrome research has been relatively low. We are extremely grateful that this is now changing; however, private foundation funding for Down syndrome research is also low and limited to certain communities. We hope that since there are many breakthrough discoveries leading to increased therapeutic potential, that diverse research and ideas are heard and supported by the community so that we can increase the nearly non-existent pharmaceutical interest in undertaking clinical trials for Down syndrome. We hope that over the next 10 years, we will see a robust increase in clinical trials and successful partnering between the patient/caregiver community, private and public grant-awarding foundations, researchers and the pharmaceutical industry so that together we can all provide an option for a higher quality of life for people with Down syndrome.

What's next for you?

In addition to my interest in the neurosciences, working in Down syndrome research has been a personal journey for me and a privilege. I hope to continue advocating for people with Down syndrome and in participating in scientific work that can benefit them in a profound way.

Reference

Aziz, N. M., Guedj, F., Pennings, J. L. A., Olmos-Serrano, J. L., Siegel, A., Haydar, T. F. and Bianchi, D. W. (2018). Lifespan analysis of brain development, gene expression and behavioral phenotypes in Ts1Cje, Ts65Dn and Dp(16)1/Yey mouse models of Down syndrome. *Dis. Model. Mech.* **11**: dmm031013.
